# Supplementary material for: Multisensory perceptual and causal inference is largely preserved in medicated post-acute individuals with schizophrenia
Source: PLoS Biol. 2024 Sep 10;22(9):e3002790. doi: 10.1371/journal.pbio.3002790 (PMC11466413; doi:10.1371/journal.pbio.3002790)
Supplement: S1 Text — (DOCX) [file pbio.3002790.s001.docx]

**S1 Text**

**Supporting methods and results**

*Analysis of response tendencies in numeric reports*

In supplemental analyses of participants’ behavioral data, we analyzed the auditory and visual numeric reports more comprehensively between groups across the combination of audiovisual signals (S1 Fig). Both HC and SCZ underestimated higher signal numbers with increasing variances, a key prediction of the scalar-variability model of numerosity estimation [1, 2]. SCZ appeared to show a stronger central tendency with stronger underestimation of large (Fig 2A) and overestimation of small numbers (Fig 2B). To quantify and compare the strength of participants’ central tendency, we modelled the influence of the auditory and visual signal number and their interaction on numeric reports with linear-logarithmic regressions (S1B Fig). Yet, slope estimates, as indicators of central tendency, did not significantly differ between both groups (S1 Table). Significant crossmodal and interaction terms indicated that observers’ numeric reports where biased by the task-irrelevant signals.

*Decoding of group membership from EEG response patterns*

In supplemental multivariate analyses of EEG data, we assessed whether schizophrenia more generally changes the multivariate ERP response patterns (i.e., grand average ERPs across electrodes) of basic sensory components and early audiovisual interactions (S7 Fig). If multivariate response patterns differed systematically between HC and SCZ, a multivariate decoder would be able to distinguish between both groups based on the response patterns [3]. Thus, we trained a linear support-vector machine classification (SVC) using LibSVM [4] to classify the diagnostic group from ERP patterns of 20 ms time windows in all but one participant. The trained SVC then predicted the group from EEG patterns of the left-out participant. In a leave-one-participant-out cross-validation scheme [3], the training-test procedure was repeated for all participants and decoding accuracy was computed as the fraction of correct classifications across all participants. The SVC’s parameter ν was optimized using a grid search within each cross-validation fold (i.e., nested cross-validation). This training-test procedure was repeated for all 20 ms time windows (i.e., 64 channels x 4 time points = 256 features) and unisensory as well as audiovisual ERP patterns. To test whether response patterns represented information on group membership (i.e., the SVC’s decoding accuracies exceeded chance level), we used a non-parametric randomization test (5000 randomizations) in which we computed the decoding accuracy of the SVC-predicted group membership as test statistic. In each randomization fold, we applied the SVC’s cross-validation scheme on randomized group membership. To correct for multiple comparisons across the EEG sampling points, we used a cluster-based correction [5] with the sum of the decoding accuracies across a cluster as cluster-level statistic and an auxiliary cluster-defining threshold of decoding accuracy = 0.55 for each time point. However, the decoder was not able to predict group membership from unisensory or audiovisual congruent ERP patterns (p > 0.05) because decoding accuracies did not exceed chance level for any time points (S7C Fig). One reason for this result was that ERP activation patterns were highly correlated across both groups (S7B Fig).

*Additional analyses that include patients with schizoaffective symptoms*

We repeated the main analyses (i.e., crossmodal bias, BCI modelling and decoding of BCI estimates from EEG data) from the main paper in a larger more heterogeneous SCZ/SCA sample by adding 6 schizoaffective (SCA) patients to the initial 17 SCZ patients. Overall, we found highly similar results in the SCZ/SCA sample (n = 23) as compared to our HC sample (n = 23). In particular, we found highly similar results for the crossmodal biases (S8 Fig and S6 Table) and the model comparison of BCI and heuristic models (S9 Fig). The between-group Bayesian model-comparison again provided strong evidence that HC and SCZ/SCA individuals relied similarly on the various decision strategies, and on model-averaging as the “winning” strategy (BF_10_ = 0.0284). The comparison of BCI model parameters between groups led to similar results (S10 Fig), except that the numeric prior’s variance was not significantly smaller for SCZ/SCA than HC (S7 Table). The correlations of BCI model parameters with PANSS positive and negative symptoms yielded also significant results (S8 Table): the visual variance correlated with positive symptoms (r = 0.585, p = 0.003) and the lapse rate marginally correlated with negative symptoms (r = 0.368, p = 0.069). Finally, decoding of BCI model numeric estimates resulted in largely comparable trajectories of decoding accuracies in HC versus SCZ/SCA groups as in our initial analyses (S11 Fig).

Overall, increasing the number of patients and hence the power of our initial analysis further corroborated our main conclusions that multisensory perceptual and causal inference was largely intact in our medicated SCZ/SCA sample. Future studies need to investigate to what extent these inference mechanisms may be affected in patients with more prominent psychotic symptoms.

**References**

1. Dehaene S. Symbols and quantities in parietal cortex: Elements of a mathematical theory of number representation and manipulation. Sensorimotor foundations of higher cognition. 2007;22:527-74.

2. Gallistel CR, Gelman II. Non-verbal numerical cognition: from reals to integers. Trends Cogn Sci. 2000;4(2):59-65. doi: 10.1016/s1364-6613(99)01424-2. PubMed PMID: 10652523.

3. Koch SP, Hägele C, Haynes J-D, Heinz A, Schlagenhauf F, Sterzer P. Diagnostic classification of schizophrenia patients on the basis of regional reward-related FMRI signal patterns. PloS one. 2015;10(3):e0119089.

4. Chang CC, Lin CJ. LIBSVM: a library for support vector machines. ACM Transactions on Intelligent Systems and Technology (TIST). 2011;2(3):27.

5. Maris E, Oostenveld R. Nonparametric statistical testing of EEG-and MEG-data. Journal of neuroscience methods. 2007;164(1):177-90.
