# Supplementary material for: Multisensory perceptual and causal inference is largely preserved in medicated post-acute individuals with schizophrenia
Source: PLoS Biol. 2024 Sep 10;22(9):e3002790. doi: 10.1371/journal.pbio.3002790 (PMC11466413; doi:10.1371/journal.pbio.3002790)
Supplement: S1 Fig — (A) Mean numeric reports (across participants mean ± SEM). (B) Mean predictions (across-participants mean) from a log-linear regression model that predicted the numeric reports from the logarithmic visual and auditory signal numbers as well as their interaction (i.e., rA/V = bA * log(nA) + bV * log(nV) + bAxV * log(nV) * log(nA) + c). Statistical results for the regression model can be found in S1 Table. (C) Mean predictions from the BCI model (across-participants mean ± SEM; model-averaging with increasing sensory variances) reproduced the logarithmic compression of numeric reports. (DOCX) [file pbio.3002790.s002.docx]

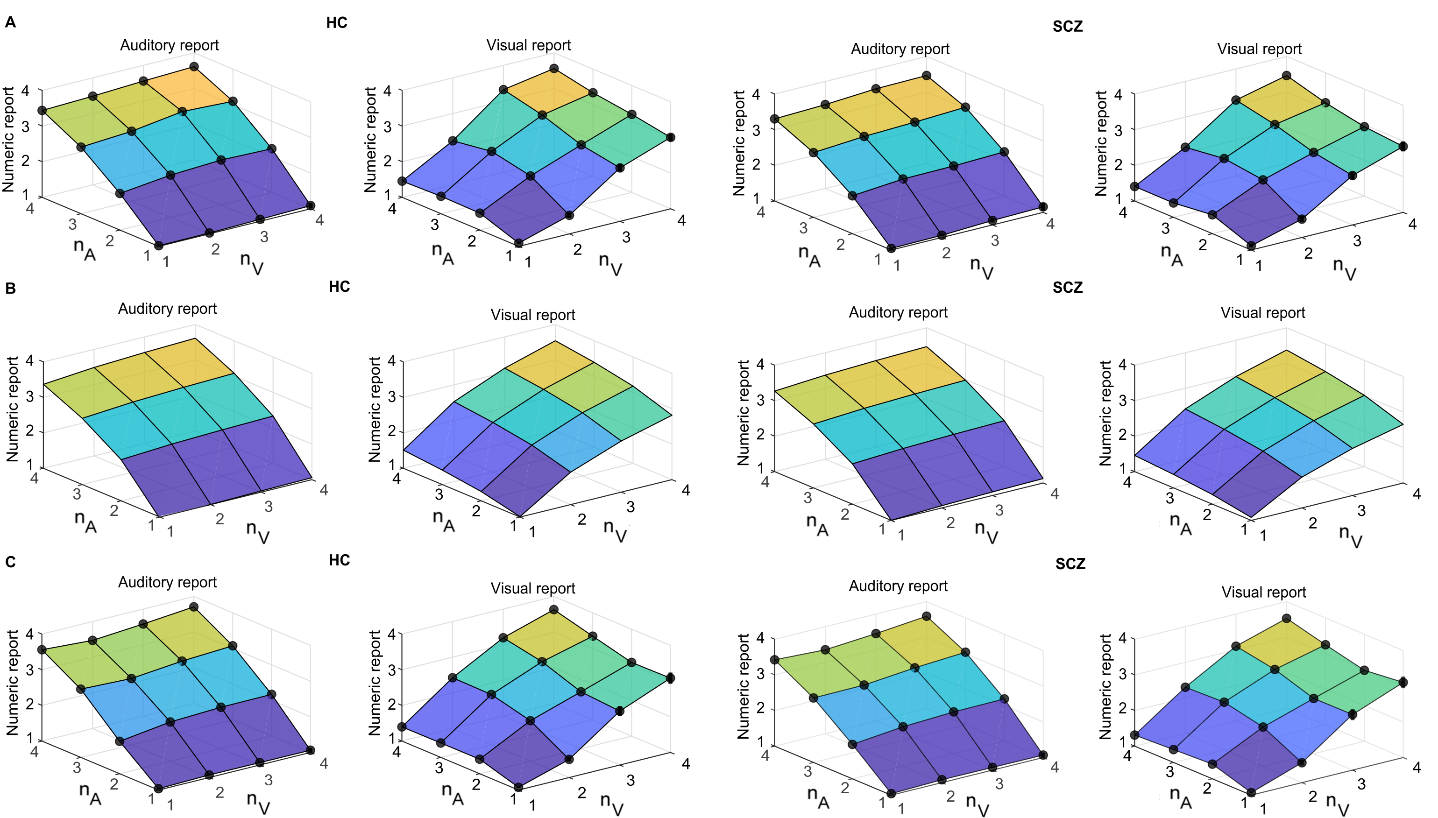


**S1 Fig. Numeric reports and predictions from a log-linear regression model as well as the BCI model as a function of the visual signal number (n_V_), auditory signal number (n_A_) and task relevance, separately for HC (left two columns, n = 23) and SCZ (right two columns, n = 17)**. **(A)** Mean numeric reports (across participants mean ± SEM). **(B)** Mean predictions (across-participants mean) from a log-linear regression model that predicted the numeric reports from the logarithmic visual and auditory signal numbers as well as their interaction (i.e., r_A/V_ = b_A_ * log(n_A_) + b_V_ * log(n_V_) + b_AxV_ * log(n_V_) * log(n_A_) + c). Statistical results for the regression model can be found in S1 Table. **(C)** Mean predictions from the BCI model (across-participants mean ± SEM; model-averaging with increasing sensory variances) reproduced the logarithmic compression of numeric reports.
