# Supplementary material for: Multisensory perceptual and causal inference is largely preserved in medicated post-acute individuals with schizophrenia
Source: PLoS Biol. 2024 Sep 10;22(9):e3002790. doi: 10.1371/journal.pbio.3002790 (PMC11466413; doi:10.1371/journal.pbio.3002790)
Supplement: S2 Fig — When fitting ex-Gaussian functions to the RT distributions, the functions’ mean (t38 = −1.325, p = 0.193, d = −0.424, BF10 = 0.620), variance (t38 = −0.511, p = 0.612, d = −0.164, BF10 = 0.346), and lambda parameter (t38 = −0.607, p = 0.548, d = −0.194, BF10 = 0.361) were not significantly different, but rather equivalent, between both groups. (DOCX) [file pbio.3002790.s003.docx]

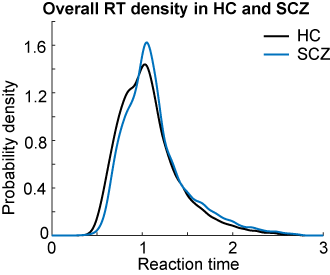


**S2 Fig. Overall reaction time (RT) density in HC and SCZ (n = 40).** When fitting ex-Gaussian functions to the RT distributions, the functions’ mean (t_38_ = -1.325, p = 0.193, d = -0.424, BF_10_ = 0.620), variance (t_38_ = -0.511, p = 0.612, d = -0.164, BF10 = 0.346) and lambda parameter (t_38_ = -0.607, p = 0.548, d = -0.194, BF10 = 0.361) were not significantly different, but rather equivalent, between both groups.
