# Supplementary material for: Multisensory perceptual and causal inference is largely preserved in medicated post-acute individuals with schizophrenia
Source: PLoS Biol. 2024 Sep 10;22(9):e3002790. doi: 10.1371/journal.pbio.3002790 (PMC11466413; doi:10.1371/journal.pbio.3002790)
Supplement: S3 Fig — The illusions were computed as difference in sensitivity (d prime, d’) between the unisensory baseline condition (V1A0 vs. V2A0) and the illusion conditions (fission: V1A2 vs. V2A2; fusion: V1A1 vs. V2A2). For the fission or fusion conditions, response “2” or “1,” respectively, were defined as signal. Thus, this illusion measure accounts for a possible shift in the response criterion which could be confounded with the fission or fusion illusions (Vanes et al, 2016). In contrast to Vanes and colleagues, a mixed-model ANOVAs did not reveal a significant difference in illusion strength between the 2 types of illusions (factor audiovisual illusion, F1,38 = 2.598, p = 0.115, part. η2 = 0.064, BFincl = 0.461) or a difference between HC and SCZ participants (factor group: F1,38 = 1.431, p = 0.239, part. η2 = 0.036, BFincl = 0.440; interaction illusion × group: F1,38 = 1.271, p = 0.267, part. η2 = 0.032, BFincl = 0.273). Note that we only included trials in which participants counted the number of flashes as ≤ 2. (DOCX) [file pbio.3002790.s004.docx]

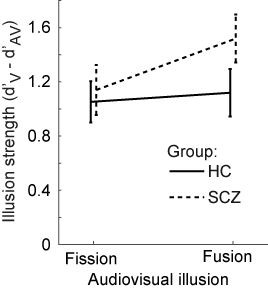


**S3 Fig. The strength of the fission and fusion illusions (across-participants mean ± SEM) is shown as a function of group (HC vs. SCZ, n = 40).** The illusions were computed as difference in sensitivity (d prime, d’) between the unisensory baseline condition (V1A0 vs. V2A0) and the illusion conditions (fission: V1A2 vs. V2A2; fusion: V1A1 vs. V2A2). For the fission or fusion conditions, response ‘2’ or ‘1’, respectively, were defined as signal. Thus, this illusion measure accounts for a possible shift in the response criterion which could be confounded with the fission or fusion illusions (Vanes et al, 2016). In contrast to Vanes et al. (2016), a mixed-model ANOVAs did not reveal a significant difference in illusion strength between the two types of illusions (factor audiovisual illusion, F_1,38_ = 2.598 p = 0.115, part. η^2^ = 0.064, BF_incl_ = 0.461) or a difference between HC and SCZ participants (factor group: F_1,38_ = 1.431, p = 0.239, part. η^2^ = 0.036, BF_incl_ = 0.440; interaction illusion × group: F_1,38_ = 1.271, p = 0.267, part. η^2^ = 0.032, BF_incl_ = 0.273). Note that we only included trials in which participants counted the number of flashes as ≤ 2.
