# Supplementary material for: Multisensory perceptual and causal inference is largely preserved in medicated post-acute individuals with schizophrenia
Source: PLoS Biol. 2024 Sep 10;22(9):e3002790. doi: 10.1371/journal.pbio.3002790 (PMC11466413; doi:10.1371/journal.pbio.3002790)
Supplement: S4 Fig — Model comparison was computed as BIC of each model relative to the fixed-criterion model with increasing sensory variance, i.e., higher is better. The model comparison included 5 models with increasing sensory variance and the 5 decision strategies (MA, model averaging; MS, model selection; PM, probability matching; FC, fixed-criterion model; SF, stochastic fusion model). The SCZ patients were rank-ordered according to their PANSS positive score on the x-axis. In cases of ties, patients were arbitrarily rank-ordered within their pair according to participant number. (DOCX) [file pbio.3002790.s005.docx]

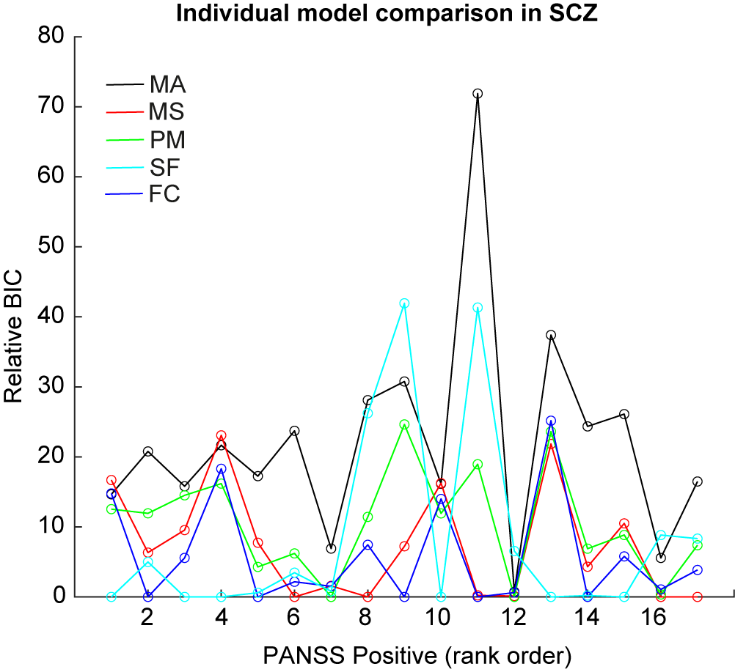


**S4 Fig. Bayesian Information Criterion values for each of the five models with increasing sensory variance are shown for each of the 17 patients in the SCZ group ranked according to their PANSS Positive score as an index of psychosis severity.** Model comparison was computed as BIC of each model relative to the fixed-criterion model with increasing sensory variance, i.e. higher is better. The model comparison included five models with increasing sensory variance and the five decision strategies (MA, model averaging; MS, model selection; PM, probability matching; FC, fixed-criterion model; SF, stochastic fusion model). The SCZ patients were rank-ordered according to their PANSS Positive score on the x-axis. In cases of ties, patients were arbitrarily rank-ordered within their pair according to participant number.
