# Supplementary material for: Multisensory perceptual and causal inference is largely preserved in medicated post-acute individuals with schizophrenia
Source: PLoS Biol. 2024 Sep 10;22(9):e3002790. doi: 10.1371/journal.pbio.3002790 (PMC11466413; doi:10.1371/journal.pbio.3002790)
Supplement: S5 Fig — (A) Data (across-participants mean ± SEM; n = 17) of the PANSS scale for positive symptoms (7 items, range 1–7) and the scale average in the SCZ sample. (B) Sum score from the LSHS-R questionnaire (12 items, range 0–48) for a subset of the HC sample (n = 5) and the SCZ sample (n = 17). The difference between both groups is significant (paired t test, t20 = −2.216, p = 0.039, d = −1.127). (C) Sum score (across participants mean ± SEM) from the PCL questionnaire (18 items, range 18–90) for a subset of the HC sample (n = 5) and the SCZ sample (n = 16). The difference between both groups is marginally significant (paired t test, t19 = −2.0271, p = 0.057, d = −1.039). Importantly, both the LSHS-R and the PCL scores varied substantially across the patient group. This allowed us to assess whether these sensitive measures of psychotic symptoms may correlate with BCI model parameters (S3 Table). PANSS: Positive and Negative Symptom Scale; LSHS-R: Launey–Slade hallucination scale; PCL: Paranoia check list. (DOCX) [file pbio.3002790.s006.docx]

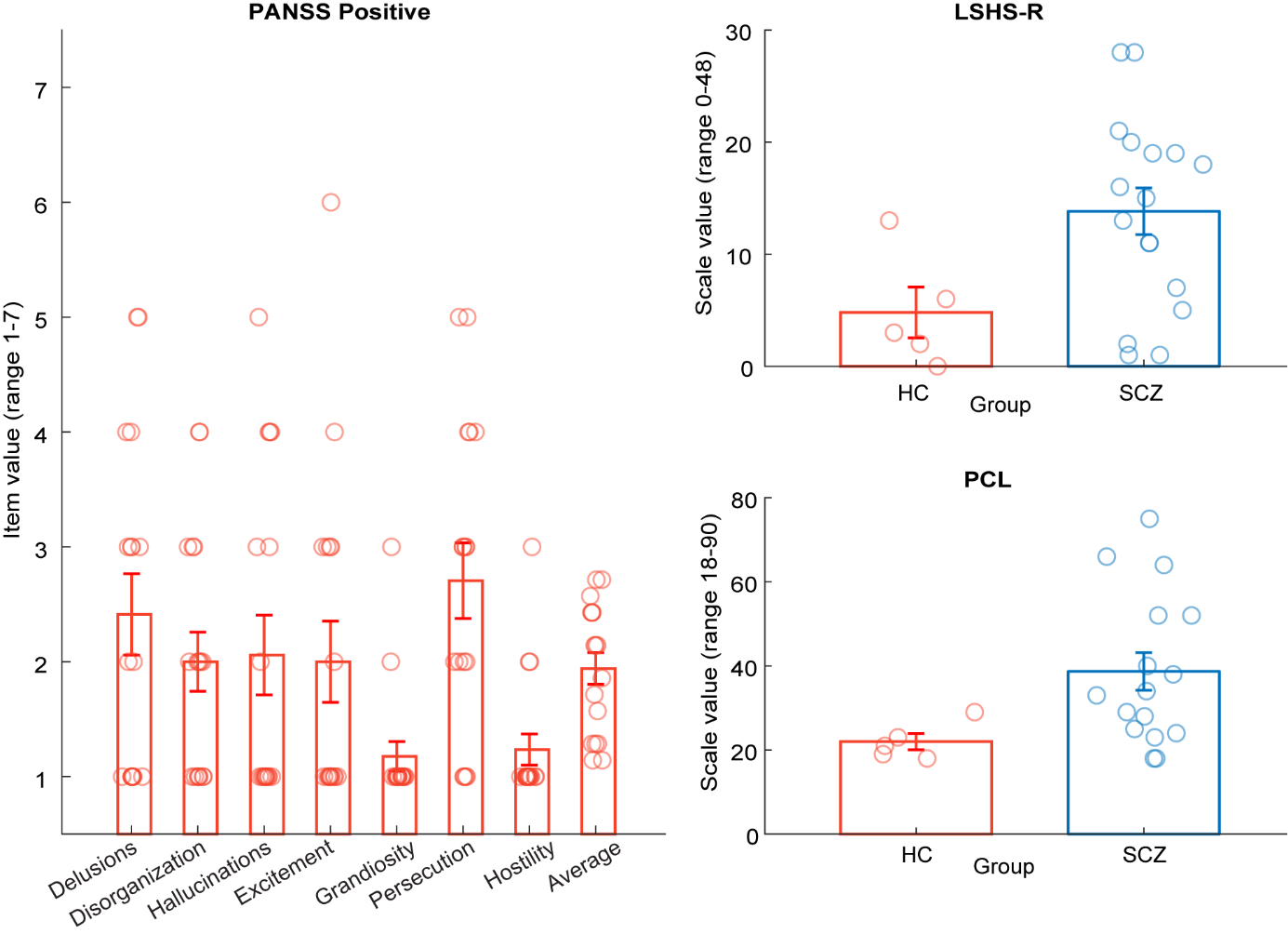


**S5 Fig. Characterization of psychosis severity.** **(A)** Data (across-participants mean ± SEM; n = 17) of the PANSS scale for positive symptoms (7 items, range 1-7) and the scale average in the SCZ sample. **(B)** Sum score from the LSHS-R questionnaire (12 items, range 0-48) for a subset of the HC sample (n = 5) and the SCZ sample (n = 17). The difference between both groups is significant (paired t-test, t_20_ = -2.216, p = 0.039, d = -1.127). **(C)** Sum score (across participants mean ± SEM) from the PCL questionnaire (18 items, range 18-90) for a subset of the HC sample (n = 5) and the SCZ sample (n = 16). The difference between both groups is marginally significant (paired t-test, t_19_ = -2.0271, p = 0.057, d = -1.039). Importantly, both the LSHS-R and the PCL scores varied substantially across the patient group. This allowed us to assess whether these sensitive measures of psychotic symptoms may correlate with BCI model parameters (S3 Table). PANSS: Positive and Negative Symptom Scale; LSHS-R: Launey-Slade hallucination scale; PCL: Paranoia check list.
