# Supplementary material for: Multisensory perceptual and causal inference is largely preserved in medicated post-acute individuals with schizophrenia
Source: PLoS Biol. 2024 Sep 10;22(9):e3002790. doi: 10.1371/journal.pbio.3002790 (PMC11466413; doi:10.1371/journal.pbio.3002790)
Supplement: S6 Fig — (A) The audiovisual crossmodal bias (CMB; across-participants mean ± SEM; n = 40) is shown as a function of a current trial’s numeric disparity (1, 2, or 3) × previous trial’s numeric disparity (0, 1, 2, 3) × task relevance (auditory vs. visual report) × group (HC vs. SCZ). A four-factorial mixed-model ANOVA including these factors revealed a marginally significant task-relevance × previous-disparity interaction (F2.1,80.9 = 2.616, p = 0.076, part. η2 = 0.064; effects of task-relevance, current numeric disparity and their interaction were p < 0.05 similarly as reported in Table 2; all other main and interactions effects, especially involving group, p > 0.05). Moreover, we assessed the more constrained hypothesis that the difference between CMB for A and V report would increase linearly with increasing numeric disparity of the previous trial: We applied contrast weights to the A-V report differences depending on the disparity at previous trial, i.e., −1.5 for 0, −0.5 for 1, 0.5 for 2, and 1.5 for 3 audiovisual numeric disparity on the previous trial. This linear contrast showed a significant linear modulation of the task relevance effect on the CMB by previous disparity (i.e., linear contrast on the interaction of previous disparity and task relevance; t114 = 2.712, p = 0.008). In other words, audiovisual interactions increased when the previous numeric disparity was small, but decreased when the previous numeric disparity was large. CMB = 1 for purely visual and CMB = 0 for purely auditory influence. (B) Visual (left panel) and auditory (right panel) numeric reports (across-participants mean ± SEM, n = 40) plotted as a function of current visual or auditory signal number, previous visual or auditory signal number and group (HC vs. SCZ). A three-factorial mixed-model ANOVA including these factors revealed a significant main effect of previous signal number (F1.7,64.7 = 33.192, p < 0.001, part. η2 = 0.466), an interaction of previous signal number and task relevance ( [file pbio.3002790.s007.docx]

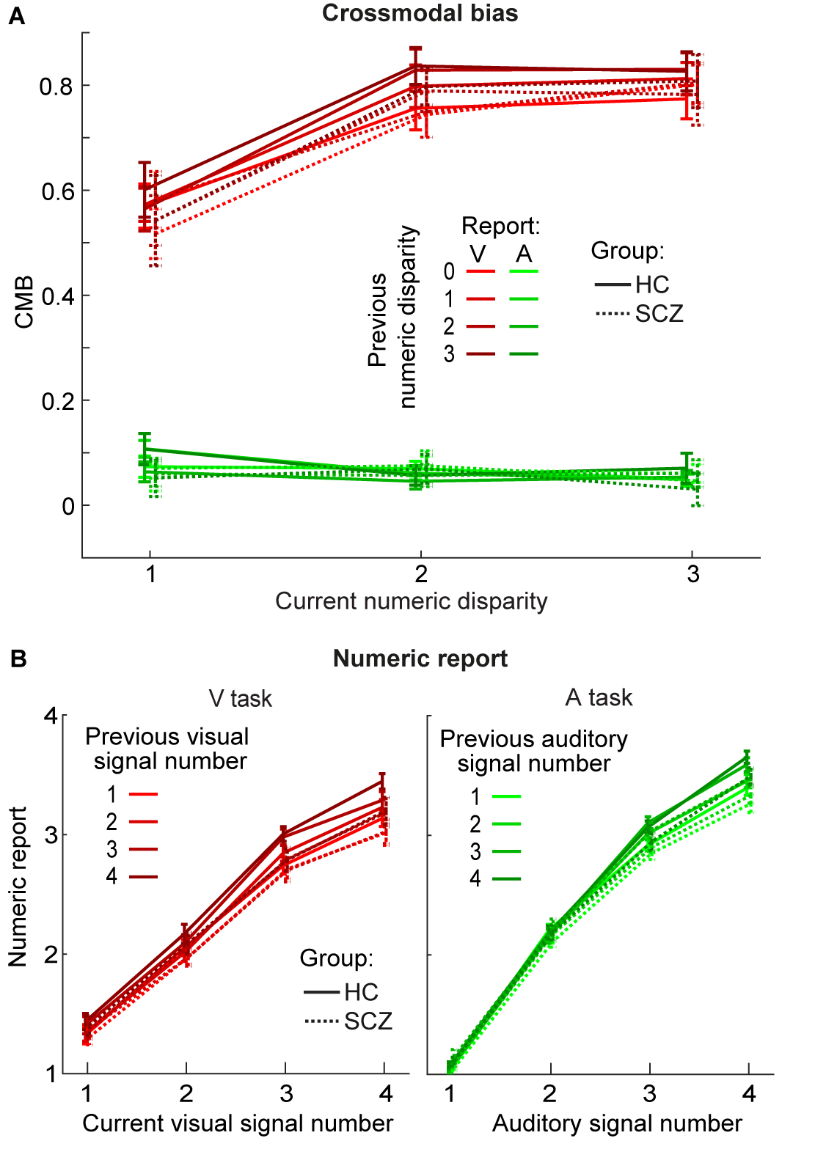


**S6 Fig. Modulation of crossmodal bias (CMB) and numeric reports by previous audiovisual numeric disparity and task-relevant signal number.** **(A)** The audiovisual crossmodal bias (CMB; across-participants mean ± SEM; *n* = 40) is shown as a function of a *current* trial’s numeric disparity (1, 2 or 3) x *previous* trial’s numeric disparity (0, 1, 2, 3) x task relevance (auditory vs. visual report) x group (HC vs. SCZ). A four-factorial mixed-model ANOVA including these factors revealed a marginally significant task-relevance x previous-disparity interaction (F_2.1,80.9_ = 2.616, p = 0.076, part. η^2^ = 0.064; effects of task-relevance, current numeric disparity and their interaction were p < 0.05 similarly as reported in Tab. 2; all other main and interactions effects, especially involving group, p > 0.05). Moreover, we assessed the more constrained hypothesis that the difference between CMB for A and V report would increase linearly with increasing numeric disparity of the previous trial: We applied contrast weights to the A-V report differences depending on the disparity at previous trial, i.e. -1.5 for 0, -0.5 for 1, 0.5 for 2 and 1.5 for 3 audiovisual numeric disparity on the previous trial. This linear contrast showed a significant linear modulation of the task relevance effect on the CMB by previous disparity (i.e., linear contrast on the interaction of previous disparity and task relevance; t_114_ = 2.712, p = 0.008). In other words, audiovisual interactions increased when the previous numeric disparity was small, but decreased when the previous numeric was large. CMB = 1 for purely visual and CMB = 0 for purely auditory influence. **(B)** Visual (left panel) and auditory (right panel) numeric reports (across-participants mean ± SEM, n = 40) plotted as a function of current visual or auditory signal number, previous visual or auditory signal number and group (HC vs. SCZ). A three-factorial mixed-model ANOVA including these factors revealed a significant main effect of previous signal number (F_1.7,64.7_ = 33.192, p < 0.001, part. η^2^ = 0.466), an interaction of previous signal number and task relevance (F_3,114_ = 3.986, p = 0.010, part. η^2^ = 0.095), and a significant interaction of previous signal number and current signal number (F_6.6,250.2_ = 8.515, p < 0.001, part. η^2^ = 0.183). The effect of current signal number and the interaction of current signal number and task relevance were also significant (p < 0.05). All other main and interactions effects, especially involving group, were not significant.
