# Supplementary material for: Multisensory perceptual and causal inference is largely preserved in medicated post-acute individuals with schizophrenia
Source: PLoS Biol. 2024 Sep 10;22(9):e3002790. doi: 10.1371/journal.pbio.3002790 (PMC11466413; doi:10.1371/journal.pbio.3002790)
Supplement: S7 Fig — (A) ERP patterns (i.e., topographies) are shown as a function of time, averaged in 100 ms time windows and separately for HC and SCZ participants. (B) Spatial correlation of multivariate ERP patterns between HC and SCZ participants as a function of time, separately for the 4 conditions (N.B.: Spatial correlations before stimulus onset might arise from expectation processes). (C) Decoding accuracy (i.e., fraction of correct classifications) of a decoder trained to classify the participant group (i.e., HC vs. SCZ) from ERP patterns of the 4 conditions. No significant clusters (p > 0.05) of decoding accuracies above the chance level of 0.5 were found in one-sided cluster-based corrected randomization tests. As a reference, the gray dashed lines indicate decoding accuracy (fraction ± 68% CI; averaged across the 4 conditions) of the decoder trained on randomized group membership (n = 5,000 randomizations). (DOCX) [file pbio.3002790.s008.docx]

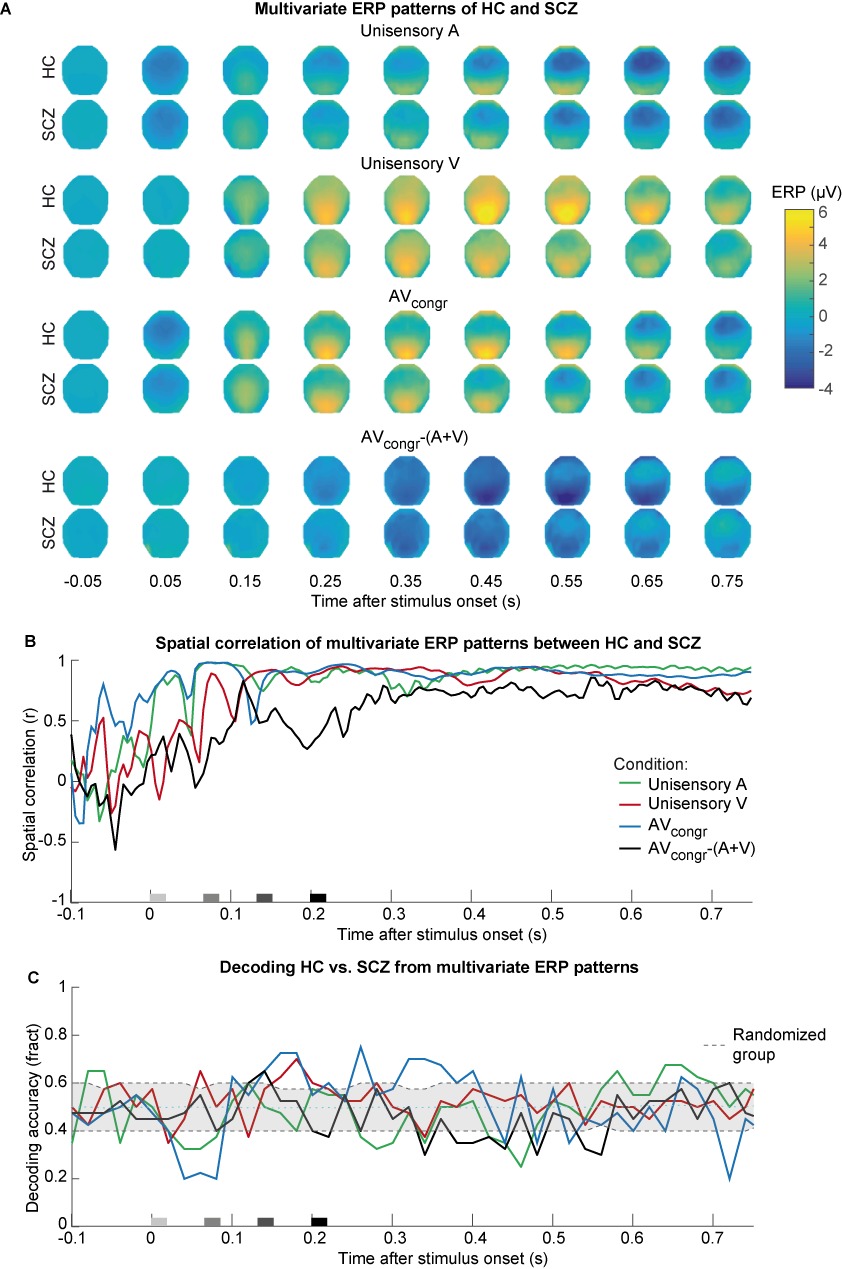


**S7 Fig. Multivariate ERP patterns, the spatial correlation of ERP patterns between HC and SCZ and decoding results (n = 40).** **(A)** ERP patterns (i.e. topographies) are shown as a function of time, averaged in 100 ms time windows and separately for HC and SCZ participants. **(B)** Spatial correlation of multivariate ERP patterns between HC and SCZ participants as a function of time, separately for the four conditions (N.B.: Spatial correlations before stimulus onset might arise from expectation processes). **(C)** Decoding accuracy (i.e., fraction of correct classifications) of a decoder trained to classify the participant group (i.e. HC vs. SCZ) from ERP patterns of the four conditions. No significant clusters (p > 0.05) of decoding accuracies above the chance level of 0.5 were found in one-sided cluster-based corrected randomization tests. As a reference, the grey dashed lines indicate decoding accuracy (fraction ± 68% CI; averaged across the four conditions) of the decoder trained on randomized group membership (n = 5000 randomizations).
