# Supplementary material for: Multisensory perceptual and causal inference is largely preserved in medicated post-acute individuals with schizophrenia
Source: PLoS Biol. 2024 Sep 10;22(9):e3002790. doi: 10.1371/journal.pbio.3002790 (PMC11466413; doi:10.1371/journal.pbio.3002790)
Supplement: S8 Fig — Participants’ audiovisual crossmodal biases (CMB; across-participants mean ± SEM; n = 46) are shown as a function of numeric disparity (1, 2, or 3), task relevance (auditory vs. visual report), and group (HC vs. SCZ). CMB = 1 for purely visual and CMB = 0 for purely auditory influence. Source data is provided in S8 Data. (DOCX) [file pbio.3002790.s009.docx]

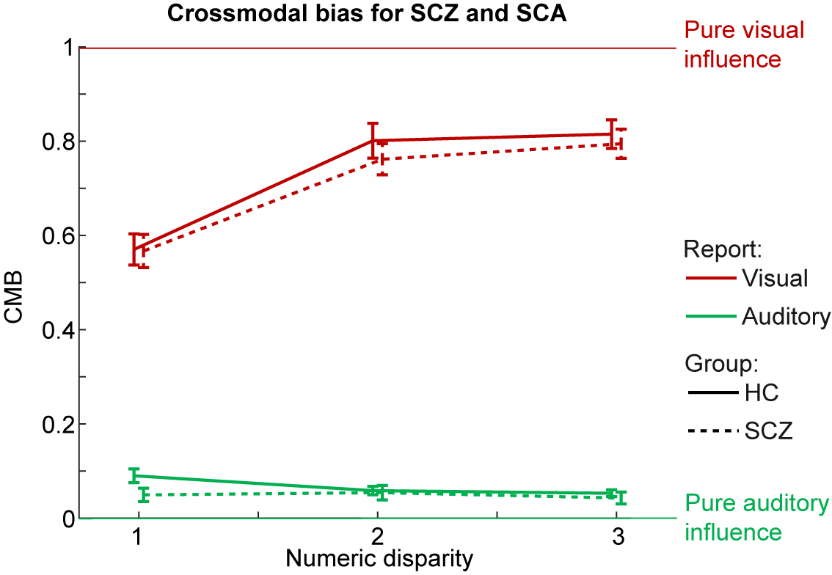


**S8 Figure. Crossmodal bias for the clinical sample including schizophrenia (SCZ, n = 17) and schizoaffective (SCA, n = 6) patients.** Participants’ audiovisual crossmodal biases (CMB; across-participants mean ± SEM; *n* = 46) are shown as a function of numeric disparity (1, 2 or 3), task relevance (auditory vs. visual report) and group (HC vs. SCZ). CMB = 1 for purely visual and CMB = 0 for purely auditory influence. Source data is provided in S8 Data.
