# Supplementary material for: Multisensory perceptual and causal inference is largely preserved in medicated post-acute individuals with schizophrenia
Source: PLoS Biol. 2024 Sep 10;22(9):e3002790. doi: 10.1371/journal.pbio.3002790 (PMC11466413; doi:10.1371/journal.pbio.3002790)
Supplement: S9 Fig — Ten models with different decision strategies, model averaging (MA), model selection (MS), probability matching (PM), fixed criterion (FC), stochastic fusion (SF), each with constant or increasing sensory auditory and visual variances. The image shows the relative model evidence for each model (i.e., participant-specific Bayesian information criterion of a model relative to the worst model summed over all participants). A larger model evidence indicates that a model provides a better explanation of our data. Source data is provided in S9 Data. (DOCX) [file pbio.3002790.s010.docx]

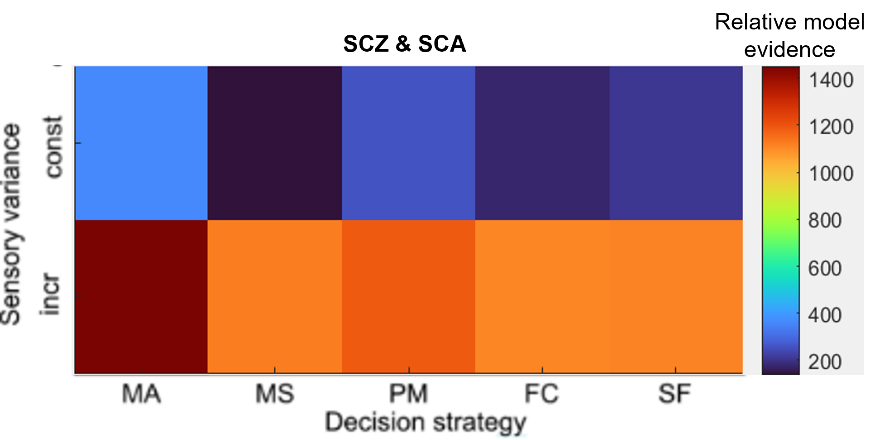


**S9 Fig. Factorial Bayesian model comparison including SCZ (n = 17) and SCA patients (n = 6).** 10 models with different decision strategies, model averaging (MA), model selection (MS), probability matching (PM), fixed criterion (FC), stochastic fusion (SF), each with constant or increasing sensory auditory and visual variances. The image shows the relative model evidence for each model (i.e. participant-specific Bayesian information criterion of a model relative to the worst model summed over all participants). A larger model evidence indicates that a model provides a better explanation of our data. Source data is provided in S9 Data.
