# Supplementary material for: Multisensory perceptual and causal inference is largely preserved in medicated post-acute individuals with schizophrenia
Source: PLoS Biol. 2024 Sep 10;22(9):e3002790. doi: 10.1371/journal.pbio.3002790 (PMC11466413; doi:10.1371/journal.pbio.3002790)
Supplement: S10 Fig — The BCI model’s decision strategy applies model averaging with increasing sensory variance. Source data is provided in S10 Data. (DOCX) [file pbio.3002790.s011.docx]

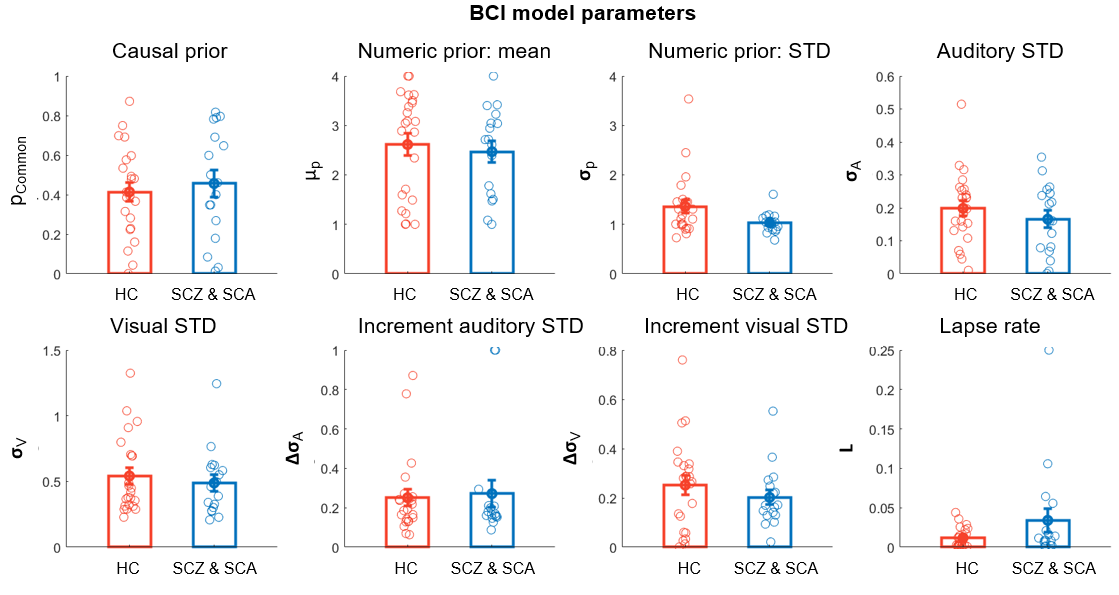


**S10 Fig. Parameters of the BCI model (across participants mean ± SEM) separately plotted for HC and SCZ (n = 17) / SCA (n = 6) patients.** The BCI model’s decision strategy applies model averaging with increasing sensory variance. Source data is provided in S10 Data.
