# Supplementary material for: Multisensory perceptual and causal inference is largely preserved in medicated post-acute individuals with schizophrenia
Source: PLoS Biol. 2024 Sep 10;22(9):e3002790. doi: 10.1371/journal.pbio.3002790 (PMC11466413; doi:10.1371/journal.pbio.3002790)
Supplement: S11 Fig — (A) Decoding accuracy (Fisher’s z-transformed correlation; across-participants mean) of the SVR decoders as a function of time and group (HC vs. SCZ & SCA). Decoding accuracy was computed as Pearson correlation coefficient between the given BCI model’s internal estimates and BCI estimates that were decoded from EEG activity patterns using SVR models trained separately for each numeric estimate. Color-coded horizontal solid lines (HC) or dashed lines (SCZ & SCA) indicate clusters of significant decoding accuracy (p < 0.05; one-sided one-sample cluster-based corrected randomization t test). (B) Bayes factors for the comparison between the decoding accuracies of HC and SCZ & SCA for each of the BCI estimate (i.e., BF10 > 3 substantial evidence for or BF10 < 1/3 against group differences). Source data is provided in S11 Data. (DOCX) [file pbio.3002790.s012.docx]

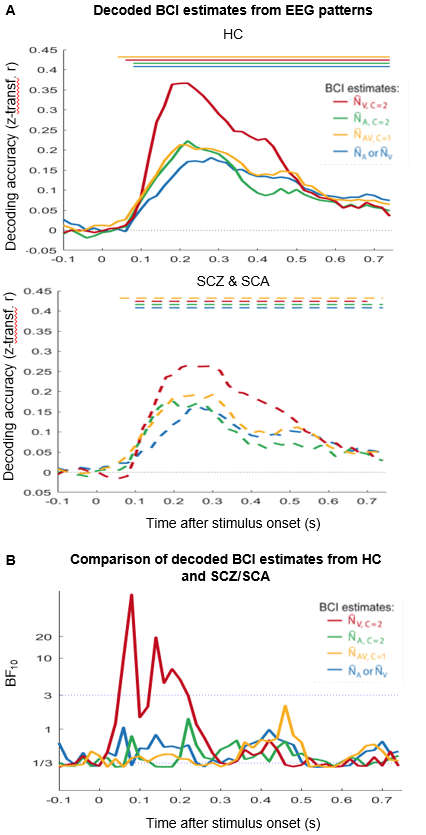


**S11 Fig. Decoding the BCI model’s numeric estimates from EEG patterns using support-vector regression (SVR) in HC versus SCZ (n = 40) / SCA (n = 6) patients. (A)** Decoding accuracy (Fisher’s z-transformed correlation; across-participants mean) of the SVR decoders as a function of time and group (HC vs. SCZ/SCA). Decoding accuracy was computed as Pearson correlation coefficient between the given BCI model’s internal estimates and BCI estimates that were decoded from EEG activity patterns using SVR models trained separately for each numeric estimate. Color-coded horizontal solid lines (HC) or dashed lines (SCZ) indicate clusters of significant decoding accuracy (p < 0.05; one-sided one-sample cluster-based corrected randomization t-test). **(B)** Bayes factors for the comparison between the decoding accuracies of HC and SCZ/SCA for each of the BCI estimate (i.e., BF10 > 3 substantial evidence for or BF10 < 1/3 against group differences). Source data is provided in S11 Data.
