# Supplementary material for: Multisensory perceptual and causal inference is largely preserved in medicated post-acute individuals with schizophrenia
Source: PLoS Biol. 2024 Sep 10;22(9):e3002790. doi: 10.1371/journal.pbio.3002790 (PMC11466413; doi:10.1371/journal.pbio.3002790)
Supplement: S12 Fig — For parameter recovery, the winning model (i.e., model averaging with increasing sensory variances) predicted responses which were then again fitted to obtain recovered parameters with the same fitting procedure as for the main analysis (i.e., initialization with 50 different random parameters; predicted distributions were generated from 5,000 simulated trials per condition). The plots show the recovered parameters as a function of the parameters originally fitted to participants’ behavioral data. The red line is a line with slope 1 and intercept 0. (DOCX) [file pbio.3002790.s013.docx]

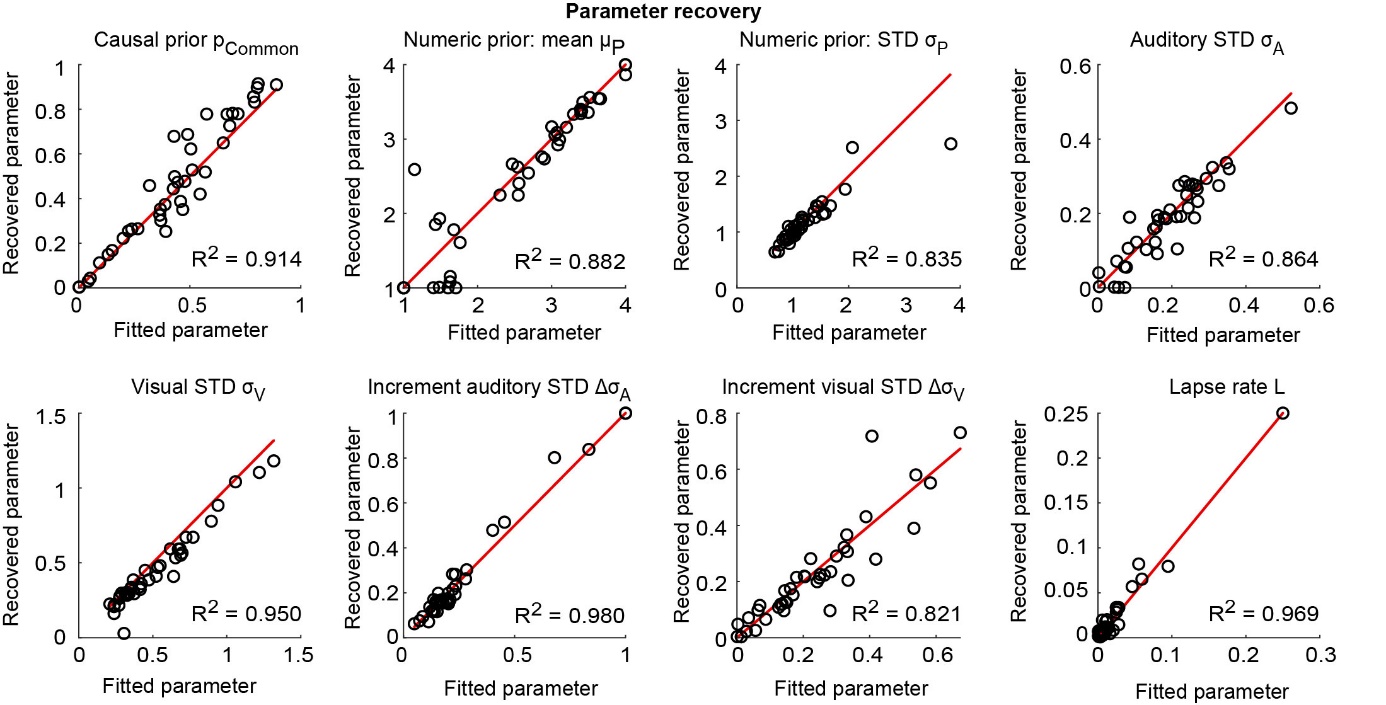


**S12 Fig. Results of the parameter recovery (n = 40).** For parameter recovery, the winning model (i.e. model averaging with increasing sensory variances) predicted responses which were then again fitted to obtain recovered parameters with the same fitting procedure as for the main analysis (i.e. initialization with 50 different random parameters; predicted distributions were generated from 5000 simulated trials per condition). The plots show the recovered parameters as a function of the parameters originally fitted to participants’ behavioral data. The red line is a line with slope 1 and intercept 0.
