# Supplementary material for: Multisensory perceptual and causal inference is largely preserved in medicated post-acute individuals with schizophrenia
Source: PLoS Biol. 2024 Sep 10;22(9):e3002790. doi: 10.1371/journal.pbio.3002790 (PMC11466413; doi:10.1371/journal.pbio.3002790)
Supplement: S13 Fig — To assess model recovery, each of the 5 models with different decision strategies (model averaging, MA; model selection, MS; probability matching, PM; fixed criterion, FC; stochastic fusion, SF) and increasing sensory variance parameters generated responses in 20 randomly selected participants. Each of the 5 models was then fitted to the generated responses of each model with the same fitting procedure as for the main analysis (i.e., initialization with 50 different random parameters; predicted distributions were generated from 5,000 simulated trials per condition). The confusion matrix shows for each generating model (rows) the fraction of participants in which a fitted model (columns) won the model comparison across the 5 fitted models based on Bayesian information criterion within an individual. The bright diagonal indicates that the generating models won the model comparisons, indicating successful model recovery. In particular, the winning model with model averaging as decision strategy was correctly recovered in 85% of the cases, thereby supporting the validity of our model-based analyses. (DOCX) [file pbio.3002790.s014.docx]

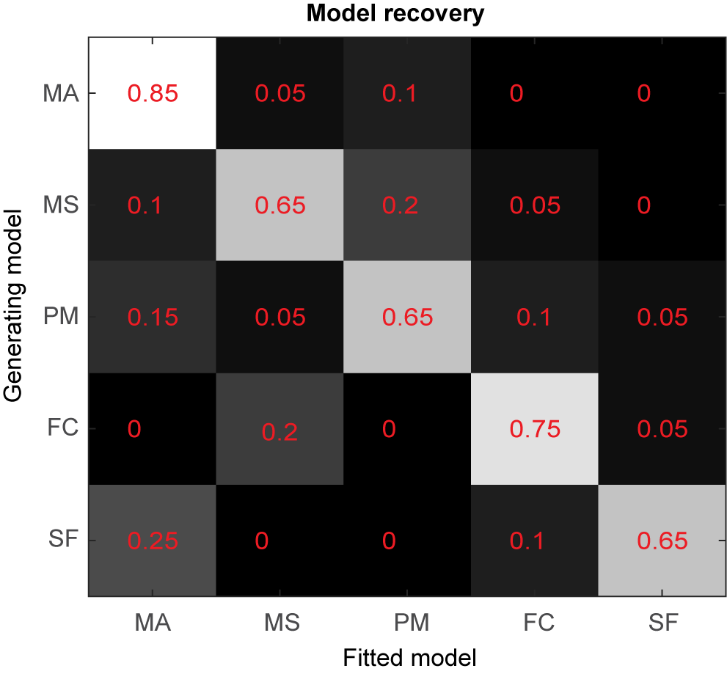


**S13 Fig. Results of model recovery.** To assess model recovery, each of the five models with different decision strategies (model averaging, MA; model selection, MS; probability matching, PM; fixed criterion, FC; stochastic fusion, SF) and increasing sensory variance parameters generated responses in 20 randomly selected participants. Each of the five models was then fitted to the generated responses of each model with the same fitting procedure as for the main analysis (i.e. initialization with 50 different random parameters; predicted distributions were generated from 5000 simulated trials per condition). The confusion matrix shows for each generating model (rows) the fraction of participants in which a fitted model (columns) won the model comparison across the five fitted models based on Bayesian information criterion within an individual. The bright diagonal indicates that the generating models won the model comparisons, indicating successful model recovery. In particular, the winning model with model averaging as decision strategy was correctly recovered in 85% of the cases, thereby supporting the validity of our model-based analyses.
