# Supplementary material for: Multisensory perceptual and causal inference is largely preserved in medicated post-acute individuals with schizophrenia
Source: PLoS Biol. 2024 Sep 10;22(9):e3002790. doi: 10.1371/journal.pbio.3002790 (PMC11466413; doi:10.1371/journal.pbio.3002790)
Supplement: S14 Fig — For parameter recovery, the model averaging model with updated priors predicted responses which were then again fitted to obtain recovered parameters with the same fitting procedure as for the main analysis (i.e., initialization with 50 different random parameters; predicted distributions were generated from 5,000 simulated trials per condition). The plots show the recovered parameters as a function of the parameters originally fitted to participants’ behavioral data. Parameters are pooled across different levels of previous trial conditions. The red line is a line with slope 1 and intercept 0. (DOCX) [file pbio.3002790.s015.docx]

*
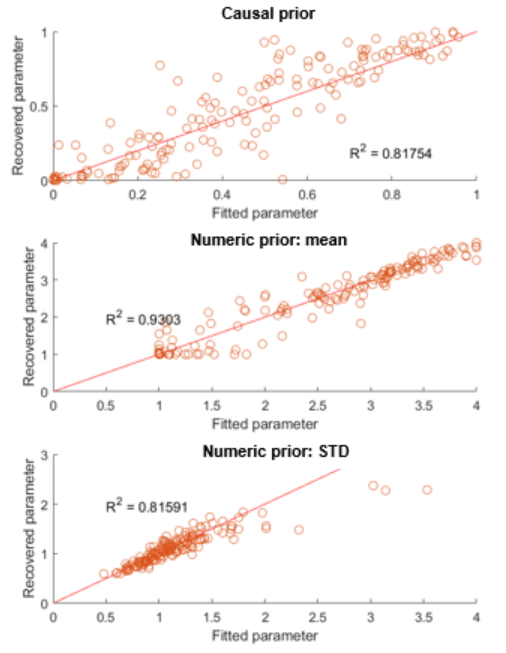
*

**S14 Fig. Results of the parameter recovery (HC & SCZ, n = 40) for the modelling analysis of updated causal and numeric priors.** For parameter recovery, the model averaging model with updated priors predicted responses which were then again fitted to obtain recovered parameters with the same fitting procedure as for the main analysis (i.e. initialization with 50 different random parameters; predicted distributions were generated from 5000 simulated trials per condition). The plots show the recovered parameters as a function of the parameters originally fitted to participants’ behavioral data. Parameters are pooled across different levels of previous trial conditions. The red line is a line with slope 1 and intercept 0.
