# Supplementary material for: Multisensory perceptual and causal inference is largely preserved in medicated post-acute individuals with schizophrenia
Source: PLoS Biol. 2024 Sep 10;22(9):e3002790. doi: 10.1371/journal.pbio.3002790 (PMC11466413; doi:10.1371/journal.pbio.3002790)
Supplement: S1 Table — (DOCX) [file pbio.3002790.s016.docx]

| **S1 Table. Statistical significance of regression parameter estimates across and between HC and SCZ in the log-linear regression model that predicted the numeric reports from the visual and auditory signal numbers as well as their interaction.** | | | | | |
| --- | --- | --- | --- | --- | --- |
| **One-sample t test on parameter estimates across HC and SCZ** | | | | | |
| Task relevance | Parameter estimates | t | p | d | BF_10_ |
| A report | b_A_ | 48.770 | <0.001 | 7.711 | >100 |
|  | b_V_ | 4.941 | <0.001 | 0.781 | >100 |
|  | b_AxV_ | 3.265 | 0.002 | 0.516 | 14.687 |
| V report | b_A_ | 6.055 | <0.001 | 0.957 | >100 |
|  | b_V_ | 18.777 | <0.001 | 2.969 | >100 |
|  | b_AxV_ | 3.406 | 0.002 | 0.539 | 20.812 |
| **Mixed-effects ANOVA on parameter estimates** | | | | | |
| Effect | F | df1, df2 | p | part. η^2^ | BF_Incl_ |
| TR | 4.853 | 1, 38 | 0.034 | 0.113 | >100 |
| PE | 185.289 | 3, 38 | <0.001 | 0.830 | >100 |
| Group | 2.250 | 1, 38 | 0.142 | 0.056 | 0.115 |
| TRxPE | 288.072 | 2.149, 81.667 | <0.001 | 0.883 | >100 |
| TRxGroup | 0.484 | 1, 38 | 0.491 | 0.013 | 0.095 |
| PExGroup | 1.448 | 3, 114 | 0.233 | 0.037 | 0.120 |
| TRxPExGroup | 0.975 | 2.149, 81.667 | 0.387 | 0.025 | 0.054 |
| Note: Regression model: r_A/V_ = b_A_ * log(n_A_) + b_V_ * log(n_V_) + b_AxV_ * log(n_V_) * log(n_A_) + c, r_A/V_ = numeric auditory/visual report, n_A_ = auditory signal number, n_V_ = visual signal number, c = constant. Parameter estimates were tested against zero across HC and SCZ using one-sample t-tests. Parameter estimates were compared between groups using a mixed-model and Bayesian ANOVA with factors TR = task relevance (auditory vs. visual report; within-participant), PE = parameter estimate from regression model (b_A_, b_V_, b_AxV_, c; within-participant) and Group (HC vs. SCZ; between-participants). Degrees of freedom are Greenhouse-Geisser corrected if sphericity is violated for an effect. | | | | | |
