# Supplementary material for: Multisensory perceptual and causal inference is largely preserved in medicated post-acute individuals with schizophrenia
Source: PLoS Biol. 2024 Sep 10;22(9):e3002790. doi: 10.1371/journal.pbio.3002790 (PMC11466413; doi:10.1371/journal.pbio.3002790)
Supplement: S2 Table — (DOCX) [file pbio.3002790.s017.docx]

| **S2 Table. Results of the Bayesian model comparison of the 2 x 5 factorial model space with factor ‘decision strategy’ and ‘sensory variance’ in HC and SCZ participants.** | | | | | | | | | | | | | | | | | |
| --- | --- | --- | --- | --- | --- | --- | --- | --- | --- | --- | --- | --- | --- | --- | --- | --- | --- |
| Decision strategy | **σ** |  | **p_common_** | **µ_P_** | **σ_P_** | **σ_A_** | **σ_V_** | Δ**σ_A_** | Δ**σ_V_** | **L** | **k** | η | **R^2^** | **relBIC** | **pEP** | **% win** |  |
| MA | c | HC | 0.47±0.05 | 2.06±0.23 | 1.80±0.23 | 0.49±0.02 | 0.79±0.06 | - | - | 0.01±0.00 | - | - | 0.87±0.01 | 385.463 | 0.00 | 0 |  |
|  |  | SCZ | 0.49±0.07 | 1.76±0.20 | 1.18±0.09 | 0.52±0.07 | 0.81±0.13 | - | - | 0.02±0.01 | - | - | 0.87±0.03 | 271.046 | 0.00 | 0 |  |
| MS | c | HC | 0.46±0.05 | 1.94±0.19 | 1.66±0.23 | 0.50±0.03 | 0.76±0.05 | - | - | 0.01±0.00 | - | - | 0.87±0.01 | 104.642 | 0.00 | 0.04 |  |
|  |  | SCZ | 0.47±0.07 | 1.78±0.20 | 1.08±0.09 | 0.52±0.07 | 0.79±0.14 | - | - | 0.02±0.01 | - | - | 0.86±0.03 | 103.438 | 0.00 | 0 |  |
| PM | c | HC | 0.44±0.05 | 2.04±0.20 | 1.59±0.22 | 0.48±0.02 | 0.73±0.05 | - | - | 0.01±0.00 | - | - | 0.87±0.01 | 212.520 | 0.00 | 0 |  |
|  |  | SCZ | 0.46±0.07 | 1.82±0.19 | 1.09±0.09 | 0.50±0.07 | 0.76±0.12 | - | - | 0.02±0.01 | - | - | 0.87±0.03 | 184.136 | 0.00 | 0 |  |
| FC | c | HC | - | 2.03±0.19 | 1.50±0.21 | 0.49±0.03 | 0.73±0.05 | - | - | 0.01±0.01 | 1.34±0.13 | - | 0.87±0.01 | 131.296 | 0.00 | 0 |  |
|  |  | SCZ | - | 1.80±0.19 | 1.09±0.09 | 0.51±0.06 | 0.82±0.16 | - | - | 0.02±0.01 | 1.16±0.18 | - | 0.87±0.03 | 135.270 | 0.00 | 0 |  |
| SF | c | HC | - | 2.05±0.22 | 1.91±0.26 | 0.47±0.03 | 0.71±0.05 | - | - | 0.01±0.00 | - | 0.22±0.03 | 0.87±0.01 | 172.262 | 0.00 | 0 |  |
|  |  | SCZ | - | 1.74±0.21 | 1.15±0.11 | 0.47±0.07 | 0.72±0.13 | - | - | 0.02±0.01 | - | 0.25±0.04 | 0.87±0.03 | 155.330 | 0.00 | 0 |  |
| MA | i | HC | 0.41±0.05 | 2.61±0.22 | 1.36±0.13 | 0.20±0.02 | 0.54±0.06 | 0.25±0.04 | 0.25±0.04 | 0.01±0.00 | - | - | 0.90±0.01 | 1452.594 | 1.00 | 0.70 |  |
|  |  | SCZ | 0.46±0.07 | 2.47±0.22 | 1.03±0.05 | 0.17±0.03 | 0.49±0.06 | 0.27±0.07 | 0.20±0.03 | 0.03±0.02 | - | - | 0.90±0.02 | 1053.740 | 0.99 | 0.65 |  |
| MS | i | HC | 0.35±0.04 | 2.50±0.20 | 1.28±0.09 | 0.22±0.03 | 0.54±0.05 | 0.26±0.05 | 0.20±0.03 | 0.01±0.00 | - | - | 0.89±0.01 | 984.443 | 0.00 | 0 |  |
|  |  | SCZ | 0.42±0.06 | 2.30±0.20 | 1.01±0.03 | 0.20±0.03 | 0.53±0.06 | 0.24±0.07 | 0.17±0.04 | 0.03±0.01 | - | - | 0.89±0.02 | 800.861 | 0.00 | 0.18 |  |
| PM | i | HC | 0.30±0.05 | 2.52±0.20 | 1.26±0.08 | 0.22±0.03 | 0.54±0.06 | 0.23±0.05 | 0.21±0.04 | 0.01±0.00 | - | - | 0.89±0.01 | 997.222 | 0.00 | 0.04 |  |
|  |  | SCZ | 0.38±0.07 | 2.29±0.21 | 1.01±0.04 | 0.20±0.03 | 0.50±0.06 | 0.25±0.07 | 0.17±0.03 | 0.03±0.01 | - | - | 0.89±0.02 | 855.084 | 0.00 | 0 |  |
| FC | i | HC | - | 2.47±0.21 | 1.29±0.10 | 0.22±0.03 | 0.55±0.07 | 0.24±0.05 | 0.25±0.06 | 0.01±0.00 | 0.85±0.13 | - | 0.89±0.01 | 981.848 | 0.00 | 0.13 |  |
|  |  | SCZ | - | 2.31±0.21 | 1.01±0.04 | 0.20±0.03 | 0.51±0.06 | 0.25±0.07 | 0.19±0.05 | 0.03±0.01 | 0.93±0.18 | - | 0.89±0.02 | 775.682 | 0.00 | 0 |  |
| SF | i | HC | - | 2.60±0.23 | 1.59±0.17 | 0.20±0.03 | 0.55±0.06 | 0.24±0.05 | 0.21±0.04 | 0.01±0.00 | - | 0.19±0.03 | 0.90±0.01 | 1048.903 | 0.00 | 0.09 |  |
|  |  | SCZ | - | 2.35±0.22 | 1.03±0.05 | 0.17±0.03 | 0.47±0.06 | 0.25±0.07 | 0.17±0.04 | 0.03±0.01 | - | 0.21±0.04 | 0.89±0.02 | 818.691 | 0.01 | 0.18 |  |
| Note: *factor decision strategies*: MA, model averaging; MS, model selection; PM, probability matching; FC, fixed-criterion model; SF, stochastic fusion model; *factor sensory variance*: c, constant sensory variance across signal numbers; i, increasing sensory variance with larger signal numbers; *model parameters*: p_common_, causal prior; µ_P_, mean of the numeric prior; σ_P_, standard deviation of the numeric prior; σ_A_, standard deviation of the auditory likelihood; σ_V_, standard deviation of the visual likelihood; Δσ increment of standard deviation per signal number; L, lapse parameter; k, fixed criterion threshold; η, probability of stochastic fusion; *model fit and comparison statistics*: R^2^, Nagelkerke’s coefficient of determination [1] using a null model of random guesses of stimulus number 1-4 with equal probability 0.25; relBIC, Bayesian information criterion at the group level, i.e. participant-specific BICs summed over all participants (BIC = LL − 0.5 m ln(n), LL = log likelihood, m = number of parameters, n = number of data points) of a model relative to the worst model (n.b. a larger relBIC indicates that a model provides a better explanation of our data); pEP, protected exceedance probability, i.e. the probability that a given model is more likely than any other model, beyond differences due to chance). % win, percentage of participants in which a model won the within-participant model comparison based on BIC. | | | | | | | | | | | | | | | | | |

**References**

1. Nagelkerke NJ. A note on a general definition of the coefficient of determination. Biometrika. 1991;78(3):691-2.
