# Supplementary material for: Multisensory perceptual and causal inference is largely preserved in medicated post-acute individuals with schizophrenia
Source: PLoS Biol. 2024 Sep 10;22(9):e3002790. doi: 10.1371/journal.pbio.3002790 (PMC11466413; doi:10.1371/journal.pbio.3002790)
Supplement: S3 Table — (DOCX) [file pbio.3002790.s018.docx]

| **S3 Table. Pearson correlations of BCI model parameters with SCZ patients‘ (n = 17) positive and negative symptoms measured by PANSS, LSHS-R and PCL.** | | | | | | | | | | |
| --- | --- | --- | --- | --- | --- | --- | --- | --- | --- | --- |
| **Scale** |  | **p_common_** | **µ_P_** | **σ_P_** | **σ_A_** | **σ_V_** | Δ**σ_A_** | Δ**σ_V_** | **L** |  |
| PANSS Positive | r | -0.057 | -0.389 | 0.047 | -0.079 | 0.625 | 0.061 | 0.058 | 0.200 |  |
|  | p | 0.825 | 0.123 | 0.873 | 0.761 | 0.003 | 0.797 | 0.841 | 0.517 |  |
|  | p_corr_ | 0.873 | 0.492 | 0.873 | 0.873 | 0.024 | 0.873 | 0.873 | 0.873 |  |
|  | r_part_ | -0.054 | -0.392 | 0.050 | -0.083 | 0.653 | 0.075 | 0.060 | 0.245 |  |
|  | p_part_ | 0.874 | 0.286 | 0.877 | 0.931 | 0.008 | 0.863 | 0.937 | 0.385 |  |
|  | p_part,corr_ | 0.937 | 0.937 | 0.937 | 0.937 | 0.067 | 0.937 | 0.937 | 0.937 |  |
| PANSS Negative | r | 0.326 | 0.184 | 0.064 | 0.074 | 0.191 | 0.343 | -0.021 | 0.483 |  |
|  | p | 0.200 | 0.486 | 0.815 | 0.770 | 0.48 | 0.180 | 0.939 | 0.029 |  |
|  | p_corr_ | 0.533 | 0.778 | 0.931 | 0.931 | 0.778 | 0.533 | 0.939 | 0.232 |  |
| LSHS-R | r | -0.105 | -0.471 | 0.147 | -0.498 | -0.205 | -0.388 | -0.015 | -0.466 |  |
|  | p | 0.679 | 0.053 | 0.584 | 0.047 | 0.45 | 0.135 | 0.956 | 0.052 |  |
|  | p_corr_ | 0.776 | 0.141 | 0.776 | 0.141 | 0.720 | 0.270 | 0.956 | 0.141 |  |
| PCL | r | -0.059 | -0.188 | 0.062 | -0.383 | 0.353 | 0.03 | -0.23 | 0.186 |  |
|  | p | 0.828 | 0.488 | 0.816 | 0.140 | 0.179 | 0.934 | 0.401 | 0.511 |  |
|  | p_corr_ | 0.934 | 0.818 | 0.934 | 0.716 | 0.716 | 0.934 | 0.818 | 0.818 |  |
| Note: Parameters from the BCI model with modeling averaging and increasing sensory variances. p_common_, causal prior; µ_P_, mean of the numeric prior; σ_P_, standard deviation of the numeric prior; σ_A_, standard deviation of the auditory likelihood; σ_V_, standard deviation of the visual likelihood; Δσ increment of standard deviation per signal number; L, lapse parameter. The significance of Pearson correlations r and partial correlations r_part_ were computed from randomization tests (n = 5000) of the correlations. For PANSS Positive, partial correlations r_part_ were computed by controlling for PANSS negative symptoms and general psychopathology. p values are provided uncorrected and p_corr_ corrected for multiple comparisons using the Benjamini-Hochberg correction for false-discovery rate. | | | | | | | | | | |
