# Supplementary material for: Multisensory perceptual and causal inference is largely preserved in medicated post-acute individuals with schizophrenia
Source: PLoS Biol. 2024 Sep 10;22(9):e3002790. doi: 10.1371/journal.pbio.3002790 (PMC11466413; doi:10.1371/journal.pbio.3002790)
Supplement: S4 Table — (DOCX) [file pbio.3002790.s019.docx]

| **S4 Table. Results of testing the decoding accuracies of the BCI estimates from EEG patterns against zero in HC, SCZ and their group difference.** | | | | | | | | | | | | |
| --- | --- | --- | --- | --- | --- | --- | --- | --- | --- | --- | --- | --- |
| BCI estimate | | HC | | | SCZ | | | | HC vs. SCZ | | | |
|  |  | time (ms) | | p | time (ms) | | p | | time (ms) | | p | |
| $\hat{N}\text{V,C=2}$ | | 60-740 | | < 0.001 | | 100-660 | <0.001 | | 120-220 | | 0.029 | |
| $\hat{N}\text{A,C=2}$ | | 80-740 | | <0.001 | | 100-600  640-700 | <0.001  0.047 | |  | | n.s. | |
| $\hat{N}\text{AV,C=1}$ | | 40-740 | | <0.001 | | 80-740 | <0.001 | |  | | n.s. | |
| $\hat{N}\text{A}$ or $\hat{N}\text{V}$ | | 80-740 | | <0.001 | | 80-740 | <0.001 | |  | | n.s. | |
| Note: The decoded BCI model’s internal estimates comprise of: i. the unisensory visual ($\hat{N}\text{V,C}\text{=2}$), ii. the unisensory auditory ($\hat{N}\text{A,C}\text{=2}$) estimates under the assumption of independent causes (C=2), iii. the forced-fusion estimate ($\hat{N}\text{AV,C}\text{=1}$) under the assumption of a common cause (C=1) and iv. the final BCI estimate ($\hat{N}\text{A}$ or $\hat{N}\text{V}$ depending on the sensory modality that is task-relevant) that averages the task-relevant unisensory and the precision-weighted estimate by the posterior probability estimate of each causal structure $\left( p(C=1 \vert x\text{A},x\text{V} \right)$). The table denotes significant clusters in one-sided (HC, SCZ) or two-sided (HC vs. SCZ) cluster-based corrected randomization t-test. n.s. denotes no significant clusters. | | | | | | | | | | | | |
