# Supplementary material for: Multisensory perceptual and causal inference is largely preserved in medicated post-acute individuals with schizophrenia
Source: PLoS Biol. 2024 Sep 10;22(9):e3002790. doi: 10.1371/journal.pbio.3002790 (PMC11466413; doi:10.1371/journal.pbio.3002790)
Supplement: S5 Table — (DOCX) [file pbio.3002790.s020.docx]

| **S5 Table. Spearman rank correlations of BCI model parameters with SCZ patients’ neurocognitive test scores (n = 17).** | | | | | | | | | |
| --- | --- | --- | --- | --- | --- | --- | --- | --- | --- |
| Scale |  | p_common_ | µ_P_ | σ_P_ | σ_A_ | σ_V_ | Δσ_A_ | Δσ_V_ | L |
| MWT-B | r | -0.3 | 0.167 | -0.244 | -0.325 | -0.353 | -0.273 | -0.054 | -0.22 |
|  | p | 0.252 | 0.513 | 0.343 | 0.202 | 0.174 | 0.291 | 0.844 | 0.389 |
| VLMT | r | -0.336 | -0.379 | -0.083 | -0.153 | -0.25 | -0.683 | -0.544 | -0.717 |
|  | p | 0.185 | 0.14 | 0.751 | 0.557 | 0.332 | 0.003 | 0.032 | 0.002 |
| TMT A-B | r | 0.397 | 0.11 | -0.181 | 0.439 | 0.515 | 0.12 | 0.074 | 0.309 |
|  | p | 0.118 | 0.666 | 0.496 | 0.082 | 0.033 | 0.65 | 0.781 | 0.232 |
| Stroop | r | -0.242 | 0.205 | -0.334 | 0.072 | 0.246 | 0.384 | 0.38 | 0.569 |
|  | p | 0.349 | 0.427 | 0.189 | 0.794 | 0.341 | 0.123 | 0.132 | 0.017 |
| Note: Parameters from the BCI model with modeling averaging and increasing sensory variances. p_common_, causal prior; µ_P_, mean of the numeric prior; σ_P_, standard deviation of the numeric prior; σ_A_, standard deviation of the auditory likelihood; σ_V_, standard deviation of the visual likelihood; Δσ increment of standard deviation per auditory or visual signal number; L, lapse parameter. The significance of Spearman rank correlations r were computed from randomization tests (n = 5000) of the correlations. Spearman rank correlations were used because of outliers in tests scores. VLMT: Verbal learning and memory test; TMT: Trail Making Test; MWT-B: Mehrfachwahl-Wortschatz-Intelligenztest (Multipe word choice intelligent test); Stroop effect: Time incongruent color word naming – time color plate naming. p values are not corrected for multiple comparisons. | | | | | | | | | |
