# Supplementary material for: Multisensory perceptual and causal inference is largely preserved in medicated post-acute individuals with schizophrenia
Source: PLoS Biol. 2024 Sep 10;22(9):e3002790. doi: 10.1371/journal.pbio.3002790 (PMC11466413; doi:10.1371/journal.pbio.3002790)
Supplement: S6 Table — (DOCX) [file pbio.3002790.s021.docx]

| **S6 Table. Main and interaction effects of task relevance (TR), disparity (Disp) and group (HC, n = 23 vs. schizophrenia, n = 17, and schizoaffective patients, n = 6) on the crossmodal bias from classical and Bayesian mixed-model ANOVAs.** | | | | | |
| --- | --- | --- | --- | --- | --- |
|  | F | df1, df2 | p | part. η^2^ | BF_incl_ |
| TR | 791.511 | 1, 44 | <0.001 | 0.947 | >100 |
| Disp | 104.715 | 1.7, 75.7 | <0.001 | 0.704 | >100 |
| Group | 0.775 | 1, 44 | 0.383 | 0.017 | 0.209 |
| TR×Disp | 104.502 | 1.6, 69.0 | <0.001 | 0.704 | >100 |
| TR×Group | 0.003 | 1, 44 | 0.954 | <0.001 | 0.051 |
| Disp×Group | 0.105 | 1.7, 75.7 | 0.873 | 0.002 | 0.086 |
| TR×Disp×Group | 1.780 | 1.57, 69.0 | 0.183 | 0.039 | 0.209 |
| Note: TR = task relevance: auditory vs. visual report; Group: HC vs. SCZ & SCA; Disp = absolute numeric disparity (1 vs. 2 vs. 3). Effects of the classical mixed-model ANOVA are Greenhouse-Geisser corrected for non-sphericity if appropriate. | | | | | |
