# Supplementary material for: Multisensory perceptual and causal inference is largely preserved in medicated post-acute individuals with schizophrenia
Source: PLoS Biol. 2024 Sep 10;22(9):e3002790. doi: 10.1371/journal.pbio.3002790 (PMC11466413; doi:10.1371/journal.pbio.3002790)
Supplement: S7 Table — (DOCX) [file pbio.3002790.s022.docx]

| **S7 Table. Comparison of BCI model parameters between HC (n = 23) and schizophrenia (n =17) / schizoaffective (n = 6) patients.** | | | | | | | | |
| --- | --- | --- | --- | --- | --- | --- | --- | --- |
|  | **p_common_** | **µ_P_** | **σ_P_** | **σ_A_** | **σ_V_** | Δ**σ_A_** | Δ**σ_V_** | **L** |
| t_44_ | -0.327 | 0.306 | 0.727 | 1.114 | 0.333 | -0.075 | 0.795 | -1.94 |
| p | 0.74 | 0.752 | 0.526 | 0.279 | 0.734 | 0.929 | 0.425 | 0.031 |
| Cohen‘s d | -0.096 | 0.09 | 0.214 | 0.329 | 0.098 | -0.022 | 0.234 | -0.572 |
| BF_10_ | 0.305 | 0.304 | 0.362 | 0.483 | 0.306 | 0.293 | 0.378 | 1.305 |
| Note: The BCI model’s decision strategy applies model averaging with increasing sensory variance. p values derived from a two-sample two-sided randomization test (n = 5000 randomizations; uncorrected for multiple comparisons). p_common_, causal prior; µ_P_, mean of the numeric prior; σ_P_, standard deviation of the numeric prior; σ_A_, standard deviation of the auditory likelihood; σ_V_, standard deviation of the visual likelihood; Δσ, increment of standard deviation per auditory or visual signal number; L, lapse parameter. | | | | | | | | |
