# Supplementary material for: Multisensory perceptual and causal inference is largely preserved in medicated post-acute individuals with schizophrenia
Source: PLoS Biol. 2024 Sep 10;22(9):e3002790. doi: 10.1371/journal.pbio.3002790 (PMC11466413; doi:10.1371/journal.pbio.3002790)
Supplement: S8 Table — (DOCX) [file pbio.3002790.s023.docx]

| **S8 Table. Correlations of BCI model parameters with patients’ (schizophrenia, n = 17, and schizoaffective, n = 6) positive and negative symptoms measured by PANSS, LSHS-R and PCL.** | | | | | | | | | | |
| --- | --- | --- | --- | --- | --- | --- | --- | --- | --- | --- |
| **Scale** |  | **p_common_** | **µ_P_** | **σ_P_** | **σ_A_** | **σ_V_** | Δ**σ_A_** | Δ**σ_V_** | **L** |  |
| PANSS Positive | r | -0.052 | -0.384 | -0.114 | -0.125 | 0.585 | 0.094 | -0.014 | 0.188 |  |
|  | p | 0.811 | 0.069 | 0.716 | 0.567 | 0.003 | 0.668 | 0.952 | 0.446 |  |
|  | r_part_ | 0.013 | -0.353 | -0.067 | -0.105 | 0.598 | 0.152 | -0.026 | 0.286 |  |
|  | p_part_ | 0.956 | 0.116 | 0.774 | 0.650 | 0.004 | 0.510 | 0.913 | 0.209 |  |
| PANSS Negative | r | 0.266 | 0.276 | 0.205 | 0.046 | -0.131 | 0.249 | -0.045 | 0.368 |  |
|  | p | 0.220 | 0.203 | 0.413 | 0.84 | 0.566 | 0.261 | 0.840 | 0.069 |  |
| LSHS-R | r | -0.051 | -0.463 | -0.001 | -0.254 | -0.014 | -0.269 | 0.124 | -0.349 |  |
|  | p | 0.828 | 0.026 | 0.996 | 0.243 | 0.951 | 0.215 | 0.578 | 0.103 |  |
| PCL | r | 0.018 | -0.144 | -0.043 | -0.259 | 0.151 | 0.075 | -0.221 | 0.160 |  |
|  | p | 0.936 | 0.517 | 0.864 | 0.242 | 0.511 | 0.802 | 0.321 | 0.483 |  |
| Note: Parameters from the BCI model with modeling averaging and increasing sensory variances. p_common_, causal prior; µ_P_, mean of the numeric prior; σ_P_, standard deviation of the numeric prior; σ_A_, standard deviation of the auditory likelihood; σ_V_, standard deviation of the visual likelihood; Δσ increment of standard deviation per signal number; L, lapse parameter. The significance of Pearson correlations r were computed from randomization tests (n = 5000) of the correlations. For PANSS Positive, partial correlations r_part_ were computed by controlling for PANSS negative symptoms and general psychopathology. The significance of partial correlations p_part_ were computed from t tests. p values are not corrected for multiple comparisons. | | | | | | | | | | |
