# Supplementary material for: Multisensory perceptual and causal inference is largely preserved in medicated post-acute individuals with schizophrenia
Source: PLoS Biol. 2024 Sep 10;22(9):e3002790. doi: 10.1371/journal.pbio.3002790 (PMC11466413; doi:10.1371/journal.pbio.3002790)
Supplement: S1 Data — (ZIP) [file pbio.3002790.s024.zip › S1_Data.docx]

**Readme of S1 Data – Figure 1**

This readme describes the data format of source data for Figure1 in Rohe, Hesse, Ehlis, Noppeney (2024) “Multisensory perceptual and causal inference is largely preserved in medicated post-acute individuals with schizophrenia”.

The data is saved as Matlab structures in .mat files which can be accessed using Matlab or Octave.

**Figure 1C-D**

- Figure 1C
  - Figure1C.respAccuracy: 40 x 2 x 2 array of Fisher-z transformed correlations
    - Dim 1 = HC participants 1-23, SCZ participants 24-40
    - Dim 2: 1 = auditory task, 2 = visual task
    - Dim 3: 1 = unisensory condition, 2 = audiovisual congruent condition
  - Figure1C.group: 1 = HC, 2 = SCZ
  - Figure1C.participantID: study ID of participant 1-40
- Figure 1D - Behavior
  - Figure1D_behavior.CMB: 40 x 2 x 2 array of crossmodal bias (CMB) from participants’ responses.
    - Dim 1 = HC participants 1-23, SCZ participants 24-40
    - Dim 2: 1 = auditory task, 2 = visual task
    - Dim 3: absolute AV numeric disparity, 1 = numeric disparity 1, 2 = numeric disparity 2, 3 = numeric disparity 3
  - Figure1D_behavior.group: 1 = HC, 2 = SCZ
  - Figure1D_behavior.participantID: study ID of participant 1-40
- Figure 1D – BCI model:
  - Figure1D_BCIModel.CMB: 40 x 2 x 2 array of crossmodal bias (CMB) computed from individual predictions of the BCI model (model averaging with increasing sensory variances)
    - Dim 1 = HC participants 1-23, SCZ participants 24-40
    - Dim 2: 1 = vuditory task, 2 = visual task
    - Dim 3: absolute AV numeric disparity, 1 = numeric disparity 1, 2 = numeric disparity 2, 3 = numeric disparity 3
  - Figure1D_BCIModel.group: 1 = HC, 2 = SCZ
  - Figure1D_BCIModel.participantID: study ID of participant 1-40
