# Supplementary material for: Multisensory perceptual and causal inference is largely preserved in medicated post-acute individuals with schizophrenia
Source: PLoS Biol. 2024 Sep 10;22(9):e3002790. doi: 10.1371/journal.pbio.3002790 (PMC11466413; doi:10.1371/journal.pbio.3002790)
Supplement: S2 Data — (ZIP) [file pbio.3002790.s025.zip › S2_Data.docx]

**Readme of S2 Data – Figure 2**

This readme describes the data format of source data for Figure 2 in Rohe, Hesse, Ehlis, Noppeney (2024) “Multisensory perceptual and causal inference is largely preserved in medicated post-acute individuals with schizophrenia”.

The data is saved as Matlab structures in .mat files which can be accessed using Matlab or Octave.

**Figure 2**

- Figure 2
  - Figure2.numericReport: 40 x 2 x 5 x 5 array of across-trial mean numeric report of each participant. Note that non-existing conditions (e.g., auditory signal number = 0 and visual signal number = 0) are coded as NaNs.
    - Dim 1 = HC participants 1-23, SCZ participants 24-40
    - Dim 2: 1 = auditory task, 2 = visual task
    - Dim 3: auditory signal number 0-4
    - Dim 4: auditory signal number 0-4
  - Figure2.group: 1 = HC, 2 = SCZ
  - Figure2.participantID: study ID of participant 1-40
