# Supplementary material for: Multisensory perceptual and causal inference is largely preserved in medicated post-acute individuals with schizophrenia
Source: PLoS Biol. 2024 Sep 10;22(9):e3002790. doi: 10.1371/journal.pbio.3002790 (PMC11466413; doi:10.1371/journal.pbio.3002790)
Supplement: S3 Data — (ZIP) [file pbio.3002790.s026.zip › S3_Data.docx]

**Readme of S3 Data – Figure 3**

This readme describes the data format of source data for Figure 3 in Rohe, Hesse, Ehlis, Noppeney (2024) “Multisensory perceptual and causal inference is largely preserved in medicated post-acute individuals with schizophrenia”.

The data is saved as Matlab structures in .mat files which can be accessed using Matlab or Octave.

**Figure 3**

- Figure 3B
  - Figure3.relativeBIC: 40 x 5 x 2 array of individual model evidences (Bayesian information criterion) relative to the individually worst model (i.e., BIC = 0).
    - Dim 1 = HC participants 1-23, SCZ participants 24-40
    - Dim 2: Model decision strategy, 1 = model averaging, 2 = model selection, 3 = probability matching, 4 = fixed criterion, 5 = stochastic fusion
    - Dim 3: sensory variance type, 1 = constant, 2 = increasing
  - Figure3.group: 1 = HC, 2 = SCZ
  - Figure3.participantID: study ID of participant 1-40
