# Supplementary material for: Multisensory perceptual and causal inference is largely preserved in medicated post-acute individuals with schizophrenia
Source: PLoS Biol. 2024 Sep 10;22(9):e3002790. doi: 10.1371/journal.pbio.3002790 (PMC11466413; doi:10.1371/journal.pbio.3002790)
Supplement: S4 Data — (ZIP) [file pbio.3002790.s027.zip › S4_Data.docx]

**Readme of S4 Data – Figure 4**

This readme describes the data format of source data for Figure 4 in Rohe, Hesse, Ehlis, Noppeney (2024) “Multisensory perceptual and causal inference is largely preserved in medicated post-acute individuals with schizophrenia”.

The data is saved as Matlab structures in .mat files which can be accessed using Matlab or Octave.

**Figure 4**

- Figure 4
  - Figure4.relativeBIC: 40 x 10 x 5 x 2 array of individual model parameters for all computational models. Note that Figure 4 plots only the model parameters from model averaging with increasing sensory variance.
    - Dim 1: 1-23 = HC participants, 24-40 = SCZ participants
    - Dim 2: Model parameters, 1 = causal prior, 2 = numeric prior mean, 3 = numeric prior STD, 4 = auditory STD, 5 = visual STD, 6 = increment auditory STD, 7 = increment visual STD, 8 = lapse rate, 9 = threshold k for fixed criterion model, 10 = eta for stochastic fusion model. Note that irrelevant parameters for a model are set to 0.
    - Dim 3: Model decision strategy, 1 = model averaging, 2 = model selection, 3 = probability matching, 4 = fixed criterion, 5 = stochastic fusion
    - Dim 4: sensory variance type, 1 = constant, 2 = increasing
  - Figure4.group: 1 = HC, 2 = SCZ
  - Figure4.participantID: study ID of participant 1-40
