# Supplementary material for: Multisensory perceptual and causal inference is largely preserved in medicated post-acute individuals with schizophrenia
Source: PLoS Biol. 2024 Sep 10;22(9):e3002790. doi: 10.1371/journal.pbio.3002790 (PMC11466413; doi:10.1371/journal.pbio.3002790)
Supplement: S5 Data — (ZIP) [file pbio.3002790.s028.zip › S5_Data.docx]

**Readme of S5 Data – Figure 5**

This readme describes the data format of source data for Figure 5 in Rohe, Hesse, Ehlis, Noppeney (2024) “Multisensory perceptual and causal inference is largely preserved in medicated post-acute individuals with schizophrenia”.

The data is saved as Matlab structures in .mat files which can be accessed using Matlab or Octave.

**Figure 5A-C**

- Figure 5
  - Figure5.modelParameters_prevTrial: 40 x 3 x 4 array of individual model parameters fitted in dependence on a previous trial’s AV disparity (for causal prior) or a previous trial’s task-relevant signal number (for numeric prior mean and STD). Note that model parameters were fitted for the BCI model with model averaging and increasing sensory variances.
    - Dim 1: 1-23 = HC participants, 24-40 = SCZ participants
    - Dim 2: Model parameters, 1 = causal prior, 2 = numeric prior mean, 3 = numeric prior STD
    - Dim 3: previous trial condition, 0-3 AV disparity for causal prior, 1-4 task-relevant signal number for numeric prior mean and STd
  - Figure5.group: 1 = HC, 2 = SCZ
  - Figure5.participantID: study ID of participant 1-40
