# Supplementary material for: Multisensory perceptual and causal inference is largely preserved in medicated post-acute individuals with schizophrenia
Source: PLoS Biol. 2024 Sep 10;22(9):e3002790. doi: 10.1371/journal.pbio.3002790 (PMC11466413; doi:10.1371/journal.pbio.3002790)
Supplement: S6 Data — (ZIP) [file pbio.3002790.s029.zip › S6_Data.docx]

**Readme of S6 Data – Figure 6**

This readme describes the data format of source data for Figure 6 in Rohe, Hesse, Ehlis, Noppeney (2024) “Multisensory perceptual and causal inference is largely preserved in medicated post-acute individuals with schizophrenia”.

The data is saved as Matlab structures in .mat files which can be accessed using Matlab or Octave.

**Figure 6A-B**

- Figure 6
  - Figure6.decodingAccuracy: 40 x 43 x 4 array of individual decoding accuracies (i.e., Fisher z-transformed correlations between true and decoded BCI estimates). Note that the comparison of decoded BCI estimates between HC and SCZ in Figure 6B computes Bayes factors from these decoding accuracies using Bayesian t-tests.
    - Dim 1: 1-23 = HC participants, 24-40 = SCZ participants
    - Dim 2: Sample points relative to AV stimulus onset
    - Dim 3: BCI model estimates, 1 = final BCI estimate ($\hat{N}\text{A}$ or $\hat{N}\text{V}$ depending on the sensory modality that is task-relevant), 2 = unisensory auditory estimate ($\hat{N}\text{A,C=2}$), 3 = unisensory visual estimate ($\hat{N}\text{V,C=2}$), 4 = forced-fusion estimate ($\hat{N}\text{AV,C=1}$)
  - Figure6.time: Time of sample points relative to AV stimulus onset in seconds
  - Figure6.group: 1 = HC, 2 = SCZ
  - Figure6.participantID: study ID of participant 1-40
