# Supplementary material for: Multisensory perceptual and causal inference is largely preserved in medicated post-acute individuals with schizophrenia
Source: PLoS Biol. 2024 Sep 10;22(9):e3002790. doi: 10.1371/journal.pbio.3002790 (PMC11466413; doi:10.1371/journal.pbio.3002790)
Supplement: S7 Data — (ZIP) [file pbio.3002790.s030.zip › S7_Data.docx]

**Readme of S7 Data – Figure 7**

This readme describes the data format of source data for Figure 7 in Rohe, Hesse, Ehlis, Noppeney (2024) “Multisensory perceptual and causal inference is largely preserved in medicated post-acute individuals with schizophrenia”.

The data is saved as Matlab structures in .mat files which can be accessed using Matlab or Octave.

**Figure 7**

- Figure 7
  - Figure7.ERP: 40 x 3 x 171 array of individual across-trial mean ERPs. Note that the contrast AV – (A + V) can be computed from this data.
    - Dim 1: 1-23 = HC participants, 24-40 = SCZ participants
    - Dim 2: Stimulus condition, 1 = unisensory auditory conditions (averaged across beep numbers 1-4), 2 = unisensory visual conditions (averaged across flash numbers 1-4), 3 = audiovisual congruent conditions (averaged across beep-flash numbers 1-4)
    - Dim 3: Sample points relative to AV stimulus onset
  - Figure7.time: Time of sample points relative to AV stimulus onset in seconds
  - Figure7.group: 1 = HC, 2 = SCZ
  - Figure7.participantID: study ID of participant 1-40
