# Supplementary material for: Multisensory perceptual and causal inference is largely preserved in medicated post-acute individuals with schizophrenia
Source: PLoS Biol. 2024 Sep 10;22(9):e3002790. doi: 10.1371/journal.pbio.3002790 (PMC11466413; doi:10.1371/journal.pbio.3002790)
Supplement: S8 Data — (ZIP) [file pbio.3002790.s031.zip › S8_Data.docx]

**Readme of S8 Data – S8 Fig**

This readme describes the data format of source data for supplemental S8 Fig in Rohe, Hesse, Ehlis, Noppeney (2024) “Multisensory perceptual and causal inference is largely preserved in medicated post-acute individuals with schizophrenia”.

The data is saved as Matlab structures in .mat files which can be accessed using Matlab or Octave.

**S8 Fig**

- S8 Fig
  - FigureS8_behavior.CMB: 46 x 2 x 2 array of crossmodal bias (CMB) from participants’ responses.
    - Dim 1 = HC participants 1-23, SCZ participants 24-46
    - Dim 2: 1 = auditory task, 2 = visual task
    - Dim 3: absolute AV numeric disparity, 1 = numeric disparity 1, 2 = numeric disparity 2, 3 = numeric disparity 3
  - FigureS8_behavior.group: 1 = HC, 2 = SCZ, 3 = SCA
  - FigureS8_behavior.participantID: study ID of participant 1-46
