# Supplementary material for: Multisensory perceptual and causal inference is largely preserved in medicated post-acute individuals with schizophrenia
Source: PLoS Biol. 2024 Sep 10;22(9):e3002790. doi: 10.1371/journal.pbio.3002790 (PMC11466413; doi:10.1371/journal.pbio.3002790)
Supplement: S11 Data — (ZIP) [file pbio.3002790.s034.zip › S11_Data.docx]

**Readme of S11 Data – S11 Fig**

This readme describes the data format of source data for supplemental S11 Fig in Rohe, Hesse, Ehlis, Noppeney (2024) “Multisensory perceptual and causal inference is largely preserved in medicated post-acute individuals with schizophrenia”.

The data is saved as Matlab structures in .mat files which can be accessed using Matlab or Octave.

**S11 Fig**

- S11 Fig
  - FigureS11.decodingAccuracy: 46 x 43 x 4 array of individual decoding accuracies (i.e., Fisher z-transformed correlations between true and decoded BCI estimates). Note that the comparison of decoded BCI estimates between HC and SCZ in Figure S11B computes Bayes factors from these decoding accuracies using Bayesian t-tests.
    - Dim 1: 1-23 = HC participants, 24-40 = SCZ & SCA participants
    - Dim 2: Sample points relative to AV stimulus onset
    - Dim 3: BCI model estimates, 1 = final BCI estimate ($\hat{N}\text{A}$ or $\hat{N}\text{V}$ depending on the sensory modality that is task-relevant), 2 = unisensory auditory estimate ($\hat{N}\text{A,C=2}$), 3 = unisensory visual estimate ($\hat{N}\text{V,C=2}$), 4 = forced-fusion estimate ($\hat{N}\text{AV,C=1}$)
  - FigureS11.time: Time of sample points relative to AV stimulus onset in seconds
  - FigureS11.group: 1 = HC, 2 = SCZ, 3 = SCA
  - FigureS11.participantID: study ID of participant 1-46
