# Supplementary material for: Multisensory perceptual and causal inference is largely preserved in medicated post-acute individuals with schizophrenia
Source: PLoS Biol. 2024 Sep 10;22(9):e3002790. doi: 10.1371/journal.pbio.3002790 (PMC11466413; doi:10.1371/journal.pbio.3002790)
Supplement: Attachment — Submitted filename: 2024_05_17_ResponseReviewer_R1_final2.docx [file pbio.3002790.s035.docx]

Thank you for your patience while your manuscript "Computations and neural dynamics of audiovisual causal and perceptual inference in schizophrenia" was peer-reviewed at PLOS Biology. Please allow me to apologize for the long delay in sending our decision, which was caused by difficulties in finding suitable reviewers and academic editors for your study. In any case, your manuscript has now been evaluated by the PLOS Biology editors, an Academic Editor with relevant expertise, and by several independent reviewers.

In light of the reviews, which you will find at the end of this email, we would like to invite you to revise the work to thoroughly address the reviewers' reports.

As you will see below, the reviewers say that the study is overall well executed and interesting. **However, all reviewers raise concerns about the small sample size, concerns about the methodological approach, and mention that the high-level claims are too strong considering the limitations of the work. Apart from the limitation of the small sample size (which should be clearly discussed), it seems that most of the concerns can be addressed with additional analyses and a more careful presentation and discussion of the previous literature and results of the current manuscript. We also encourage you to try to make your paper as accessible as possible to readers who are not familiar with the Bayesian modeling procedures.**

Given the extent of revision needed, we cannot make a decision about publication until we have seen the revised manuscript and your response to the reviewers' comments. Your revised manuscript is likely to be sent for further evaluation by all or a subset of the reviewers.

We expect to receive your revised manuscript within 3 months. Please email us ([plosbiology@plos.org](mailto:plosbiology@plos.org)) if you have any questions or concerns, or would like to request an extension.

At this stage, your manuscript remains formally under active consideration at our journal; please notify us by email if you do not intend to submit a revision so that we may withdraw it.

*We thank the editor and the reviewers for all their efforts to review and evaluate our manuscript and their constructive comments. We are pleased about their overall positive evaluation. Based on their comments, we put an enormous amount of efforts into improving the manuscript. In particular, we added several novel and control analyses, acknowledge the limitations of our study, include a careful discussion of our results and rewrote substantial parts of the manuscript to enhance its accessability to non-expert readers (e.g., in the Introduction, p. 4 l.7ff, and Results, p. 10 l. 31ff ). We feel that our revisions have substantially improved our manuscript.*

*We have highlighted all changes in the manuscript (in yellow) and refer the reviewers to changes in the manuscript (page/line) in our responses.*

Reviewer #1: This article investigates audiovisual perception in a sound-induced flash illusion paradigm using behavioral, computational (Bayesian modelling) and neuroimaging analysis in 17 SCZ patients as compared to 23 controls. The results are essentially negative: the authors found comparable behavioural, computational and neural mechanisms/ EEG signatures in SCZ and HC and no difference in the model parameters or winning model. Bayesian inference and more particularly causal inference has recently been proposed to be at the origin of differences in SCZ (as well as ASD) so the line of work is important.

Though dense, the article is very clearly written. It is using a 1-4 beeps x 1-4 flash factorial experimental design which is interesting, the computational analysis is thorough and the EEG decoding exercise interesting.

*Answer: Thanks for the overall positive evaluation of our manuscript and the helpful comments.*

In my understanding (apologies if I have missed relevant information), the paper seems to suffer from a few limitations however, which may limit its impact and significance:

- One obvious issue with the study is the sample size. 17 SCZ is a small sample. I couldn't find a power analysis to motivate the sample size of 17 (some indication for supporting a somewhat larger sample size of the 26 initial participants). As is common for such clinical studies, the SCZ group is also very heterogeneous both in terms of comorbidities and treatment. The SCZ group has been medicated for a long time and stable with low PANSS positive scores. It is also apparent in the modelling that the individual parameters are very widespread.

*A: We determined the sample size based previous studies reporting multisensory integration deficits in schizophrenia which ranged from 12 to 30 patients per group (de Gelder et al., 2003; Pearl et al., 2009; Williams et al., 2010). Further, a sample size of 25 participants per group yields a statistical power 0.97 for a large effect size (d = 1.00). Please note that our initial patient sample included 26 patients. However, to make our sample more homogeneous we excluded 6 patients because they fulfilled the criteria for schizoaffective disorder and substance-induced psychotic disorder. Further, two patients had to be excluded, because they appeared to have reported the number of beeps across all tasks.*

*Following reviewer’s 2 suggestions we have now included the results of a new analysis that also included the schizoaffective patients. Importantly, this new analysis largely replicated our initial findings that patients showed a remarkably intact behavioral and neural response profile. However, we agree with the reviewer that a larger number of patients would have been beneficial. Our revised paper now reports this additional analysis i.e. with 23 patients in the Supporting results (p. 2; Fig. S8-S11; Tab. S6-S8).Further, we clearly acknowledge and discuss the limited sample size in the discussion (p. 20, l. 15).*

*We also agree with the reviewer that even when limiting our sample only to patients with schizophrenia, the remaining heterogeneity in comorbidities and treatment may have obscured changes in multisensory causal inference in SCZ. Most importantly, our sample mainly included chronic SCZ patients with low acute psychotic symptoms. Thus, our findings may not generalize to acute psychotic and/or unmedicated patients (see also our comments to reviewer 2, who also raised this point).*

*We now discuss in-depth the limitations due to sample size, patients’ medical heterogeneity and low psychosis-acuity in a Discussion paragraph (p. 20, l. 15ff, limitation points 1-3).*

- It is also unclear whether the experimental design is well chosen to identify differences in SCZ. The SOA is not varied (and the choice of SOA is not very clear motivated either) which could perhaps explain the negative results, in particular if the differences are about the temporal binding window. I think better motivation with respect to previous literature and experimental design and maybe replication of conditions where differences in schizophrenia were found would have helped.

*A: The current study presented only synchronous AV stimuli (i.e., SOA = 0). Instead, we manipulated the number of flashes and beeps to assess how* ***numeric disparity*** *influences audiovisual binding – thereby following our own and previous research on causal inference* (Odegaard et al., 2016; Odegaard & Shams, 2016, 2017; Rohe et al., 2019)*. We agree with the reviewer that future research may instead focus on the neural mechanisms that instantiate the temporal binding window by manipulating the SOA rather than the number of sensory signals.*

*Indeed, elegant past research has revealed difference in temporal binding windows between SCZ and autism using audiovisual simultaneity judgments tasks* (Noel et al., 2018; Zhou et al., 2018)*. It is important to recognize that studies presenting only one or two flashes with two beeps at variable SOAs would not be powerful to study whether SCZ and controls differ in how they read out their final estimate from the posterior distribution (e.g. decision strategies of model selection, model averaging or probability matching), which was a key question of the current study. Investigating decision strategies required to manipulate numeric disparity over a broad range. Conversely, adding the factor SOA to our current study would have resulted in far too many conditions.*

*In short, experimental paradigms that manipulate either the number of flashes/beeps (as our study) or the SOA are optimized to address different questions.*

*We now motivate the experimental design more clearly in the Results section (p. 6, l. 15). In the Discussion, we also highlight why we manipulated numeric disparity and suggest future research assessing the neural mechanisms of the temporal binding window (p. 21, l.9).*

- For the modelling part, Figure S5 seems to be showing parameter recovery for the winning model rather than model recovery (as indicated in the caption?). It is unclear then what the performance is in terms of model recovery? Similarly, it is not very clear whether the winning is actually a good model for the data (Wilson & Collins, 2019).

*A: Many thanks for pointing out this error. Figure S12 (formerly S5) obviously shows parameter recovery, and not model recovery. We corrected this error. Following the reviewer’s important suggestions we have now also performed model recovery for our five Bayesian / heuristic models as suggested by* Wilson & Collins (2019) *as well as* Acerbi et al. (2018)*. For computational expediency, we focussed on the five models with increasing sensory variances that clearly won the model comparison compared to fixed sensory variances (Fig. 3). The confusion matrix indicated that the models were identifiable with reasonable accuracy, confirming the reliability of our model fitting procedure (Supporting Fig. S13).*

*We added the results for model recovery to the Methods section (p. 30, l. 1):*

- Some aspects are unclear in the text, e.g. are the behavioural effects that are significant (numeric prior and visual variance) corrected for multiple comparisons?

*A: The p-values for the comparison of BCI model parameters (Tab. 3) and the correlation of visual variance with positive symptoms were not corrected for multiple comparison. When using Benjamini-Hochberg correction of the false discovery rate across the 8 parameters, the difference in numeric prior is not significant (p_corr_ = 0.210). We acknowledge this limitation in the results (Tab. 3 and text, p. 12, l. 16) and the Discussion (p. 17, l. 29). Please note that we have carefully interpreted this finding already in our previous version. The correlation between visual variance and positive symptoms survived the multiple-comparison correction across the 8 parameters at p < 0.05, which we now report in the results section (p. 12, l.20 and Supporting Tab. S3).*

- Page 12: is the SVM trained on both HC and SCZ data, together or separately? The hypotheses being tested here could be clearer.

*A: The SVM decoders were trained individually in each participant, which implies that the decoders were also trained separately for HC and SCZ. It is generally recommended to train SVR models individually in each participant, because the activation patterns may not generalize across participants. This approach enables us to investigate whether the strength and/or time courses of neural representations that encode the BCI model’s numeric estimates were shared or different between the two groups. In other words, while our results show that the time courses of the neural representations encoding the BCI model’s estimates generalize across participants, they do not imply that the activation patterns per se generalize across participants. We explain the decoding approach and the hypothesis more clearly in the results section (p. 15, l. 15).*

- "In both HC and SCZ, the decoders predicted the BCI estimates from EEG patterns significantly better than chance over most periods of the post-stimulus time window" "Significantly better than chance" is vague, it is frustrating to have to go to the figure to have an idea how well the decoding is doing and it is unclear what level of accuracy would be necessary for making clear conclusions.

*A: Following the reviewer’s criticism, we have now explained “chance level” (p. 15 l. 19) and also directly report the “decoding accuracy” in the Results section (p. 15, l. 23).*

**Reviewer #2:** Rohe et al. use an EEG-compatible multimodal sensory-integration task to evaluate causal Bayesian inference in medicated patients with schizophrenia and healthy controls. The authors then use computational modeling and EEG decoding to characterize behavioral and neural computations underlying various levels of inference. Overall, they conclude that neither behavioral nor neural data suggest alterations in inference are present in medicated patients with schizophrenia. This article presents a compelling experimental framework and several interesting analyses. Overall, the experimental design and analytical techniques are of excellent quality, particularly for a clinical study.

Despite my general enthusiasm, however, I have concerns about the characterization of prior literature, interpretation of the results, and some analysis decisions that should be addressed before I can make a recommendation for publication.

In general terms, and despite the obvious quality of this work, it is not clear what the main takeaways and contributions to this literature are. Given the sample characteristics (see below), I believe it is fair to say that this study is not well positioned to rule in/out prior over/underweighting as a model of psychotic states, and therefore it cannot resolve mixed findings in this literature. The paper's contributions should be made clearer (novel methods and proof-of-concept application to this area?) and the conclusions should be very explicitly qualified with these limitations in mind (particularly in the abstract and the discussion sections, and with particular attention to the strength of evidence for the null hypothesis and generalizability of these conclusions).

*A: Thank you! Following the reviewer’s suggestions we have now clarified the key contributions of this paper (p. 18, l. 14; p. 20, l. 5) as well as its limitations (p. 20 l. 15). Indeed, our sample of patients with predominantly negative symptoms is not well positioned to assess the role of prior weighting in psychosis. In the Discussion, we now emphasize the study’s strengths while fully acknowledging its limitations concerning the sample size and patient characteristics such as low psychosis severity in our sample (p. 20, l. 24). In the abstract, we also limit our finding to post-acute medicated patients (p. 2, l. 14).*

Major concerns

1.	Throughout, the authors treat schizophrenia and psychosis interchangeably, from presentation of the literature to analytical justification. This is problematic. Strong/precise prior models (e.g., Powers et al., 2017) are models of psychosis severity and not models of schizophrenia. Importantly these models assume state-dependence of neurocognitive phenotypes of psychosis (Kafadar et al., 2022). So it is critical to separate state-dependent effects from trait-like effects in schizophrenia patients that could be linked to negative or cognitive symptoms, and which have been associated with distinct, even opposite mechanisms from those associated selectively with positive symptoms (e.g., see work from Rick Adams). This confusion arises throughout the manuscript (specific comments below).

*A: Thank you for raising this important issue. We agree that the distinction between psychosis and schizophrenia as well as the dissociation between trait- and state-dependent effects is crucial. In the revised version, we clarify that our study investigates perceptual inference in patients with chronic schizophrenia, prominent negative symptoms but low scores in hallucinations. We have rewritten large parts of the introduction (p. 3, l. 12) and revised all parts of the manuscript accordingly.*

a.	Description of previous literature: The discussion of the link between hallucinations and prior weighting (citations 8-17) does not distinguish between findings that compare patients vs. controls and studies that consider psychosis (or psychosis-like) severity. Overall the balance of the literature presented gives the wrong impression. The majority of the literature points to a positive relationship between psychosis severity and prior weighting, even if a minority of studies obtained conflicting findings. The literature review should be balanced in terms of the evidence for prior overweighting in patients with more severe psychotic symptoms (hallucinations more concretely) vs. evidence against this proposition. Again, this should separate psychotic states/severity versus trait-like schizophrenia phenotypes (the latter of which, but not the former, are associated with cognitive deficits and amotivation, which can broadly affect cognitive performance). There should also be

a distinction between clinical and non-clinical populations.

*A: Thank you. We have now carefully balanced the discussion of previous findings and differentiate between psychotic states vs. chronic traits in the introduction (p. 3, l. 12) and in the Discussion (p. 18, l. 10; p. 20, l. 26), where we also now differentiate between clinical and non-clinical populations (p.20, l.34).*

b.	Data analysis: In general, patient vs control comparisons are tested when analyses across psychosis severity (with particular emphasis on hallucination severity) would also be appropriate. There should also be a clearer justification for the scales and items used in the correlations with psychosis and there should be a clearer description of the distribution of psychosis severity in this sample, with emphasis on whether this small sample has enough variability to really test psychosis-related models which require sufficient range of psychosis severity.

*A: Following the reviewer’s suggestion, we have added justifications for scales and items in the Methods (p. 23, l. 20) and now report –for the clinical sample- the distribution of individual PANSS positive items as well as further hallucination (LHSH) and delusion scores (PCL) from self-reported questionnaires in supplemental Fig. S5 and averages in Tab. 1. LSHS and PCL assess subclinical or prodromal hallucinatory and delusional symptoms and, therefore, also capture psychosis severity at the lower end of the psychosis continuum. As assumed by the reviewer, especially PANSS positive items showed a small variability and floor effects in the SCZ sample. LHSH and PCL data was more variable and, compared to the small subset of HC (n=5), showed significantly more hallucinatory experiences and paranoid thinking. Given the higher variability, LSHS and PCL may be a more sensitive measure of psychosis severity than PANSS positive scores that only resolve positive symptoms above the clinical threshold. Yet, when we correlated LSHS and PCL scores with BCI model parameters in the SCZ sample (Supporting Tab. S3), we did not find additional strong correlations of BCI parameters with psychosis severity. Only the LHSH score was marginally (uncorrected) and negatively correlated with the auditory variance. Possibly, the small sample size may have limited the statistical power to obtain significant correlations with model parameters for this putatively more psychosis-sensitive measure. We report the correlation of BCI parameters and LSHS as well as PCL in the Results (p. 12, l. 22) and supporting Table S3. In the Discussion, we elaborate on the correlation between sensory variances and positive symptoms (p. 17, l. 27). Because we did not systematically collect data on psychosis symptoms in the non-clinical sample (LSHS and PCL were given only to 5 HCs), we cannot perform analyses along the continuum of psychosis severity including controls and SCZ patients.*

c.	Results interpretation: This data doesn't appear to argue against strong prior models of psychosis but rather suggests that inference processes are largely spared in a population of medicated schizophrenia patients with low psychosis severity. To the contrary, the authors do report effects of prior variance that relate to psychosis, consistent with many findings in the literature.

i.	In the discussion, the authors note that "we cannot exclude the possibility that SCZ may have been less vigilant … which could be misinterpreted as overreliance on their numeric prior." Wouldn't the authors suspect that individual differences in vigilance would be captured by the lapse term? Which would be consistent with the finding that this term correlates with negative symptoms? To clarify the specificity of the identified relationships between prior variance and positive symptoms, negative and general symptoms should be statistically controlled for in these analyses.

*A: Reduced vigilance and/or reduced motivation (i.e. task compliance) may lead to biased results depending on the adopted response strategy of a participant in a state of low vigilance/motivation: a) A low-vigilant/motivated participant may preferably report a specific number of signals (e.g., ‘2’), thus leading to a consistent bias towards a specific signal number. This would be captured by a stronger numeric prior, i.e. lower numeric prior variance. b) A low-vigilant/motivated participant may report a random number of signals according to a uniform distribution, and this response pattern is captured by the lapse rate modelling an additive uniform distribution on top of the BCI model’s response distribution.*

*Because we did not find a significantly larger lapse rate in the SCZ group, we previously concluded that at least at group level a random-response strategy is unlikely. However, we agree with the reviewer that the correlation between lapse rate and negative symptoms (p. 13, l. 2 and supporting Tab. S3) may also indicate that higher vigilance (or rather lack of motivation which is part of negative symptoms) leads to more random response behaviour. We clarify the distinction between both response strategies in the Discussion section (p. 18, l. 1).*

*Following the reviewer’s important suggestion, we controlled the correlation between visual variance and positive symptoms for negative symptoms and general psychopathology using a partial correlation approach. The reported correlation was unaffected by this statistical control. We report this in the results section (p. 12, l. 20 and Supporting Tab. S3).*

2.	Sample: The sample is relatively small which should be acknowledged when arguing for the null hypothesis. The sample is also possibly not representative of the population of patients with schizophrenia as a whole, particularly in early or untreated states. The patients have relatively high doses of antipsychotics, low psychosis severity, and are largely cognitively intact. Furthermore, the recruitment strategy/recruitment sources is not discussed, which may be relevant to characterizing this cohort. Some of these points are acknowledged in the discussion but should be clarified during key takeaway statements throughout the manuscript. This is especially important since the authors seem to be arguing that the results are contradictory with the rest of the literature.

*A: Our previous manuscript already acknowledged these limitation. Following the reviewer’s concerns, our revised version emphasizes these points even more strongly. We included a limitations paragraph in the discussion (p. 20, l. 15; see also response to Reviewer 1 above).*

*Further, we agree that our sample of participants is relatively strongly medicated, has low acute psychosis severity (Supporting Fig. S5) and is largely cognitively intact (Tab. 1). We discuss this constrained representativeness of our sample (implied by the recruitment process) in the limitations paragraph in the discussion (p. 20, l. 24; see also response to Reviewer 1 above).*

*Further, we elaborate on the recruitment process in the Methods (p. 22, l. 10)*

a.	Schizoaffective patients were excluded in this analysis which is very uncommon in this literature. Given that including them would substantially increase the sample size I would like to see analyses including these patients.

*A: We excluded schizoaffective (SCA) patients to reduce the clinical heterogeneity of the sample. When including the 6 SCA patients into the clinical sample (n = 23 overall), we overall found highly similar results which we now mention in the Results (p. 17, l. 30), Discussion (p. 20, l. 18), the Methods (p. 22, l. 26) and the Supporting Methods and results (p. 2). Specifically, we found highly similar results for the CMB (Supporting Fig. S8 and Tab. S6), the BCI model comparisons (Fig. S9), the comparison of BCI model parameters between the clinical (SCZ and SCA) and the HC samples (Fig. S10 and Tab. S7), the correlation of BCI model parameters and positive/negative symptoms (Tab. S8) and well as for decoding accuracies of BCI model estimates (Fig. S11).*

*Importantly, the stronger numeric prior in SCZ patients was no longer significantly lower compared to HC when we included also SCA patients. Therefore, we raise a cautionary note about this in the Results (p. 12, l. 16) and Discussion (p. 17, l. 30).*

3.	The authors evaluate evidence for trial-by-trial learning by splitting the data into subsets with different trial histories and fitting the BCI model to these subsets. This approach is not standard and should be more carefully validated and supported. The authors should first demonstrate evidence for these effects using model-agnostic approaches (e.g., lagged-regression predicting report as a function of previous trial identity), showing that their model-based results capture qualitative features of the raw data. As it stands, it is unclear whether there are even meaningful trial-history effects on this task, or whether they should be expected based on this literature. Second, they should demonstrate that this fitting approach produces valid and interpretable parameter estimates via a parameter recovery. Alternatively, there are models in the literature that dynamically update priors as a function of trial-by-trial evidence. A model with this structure would be a more

appropriate test of this question. That said, an alternative would be to eliminate this section altogether, since I am not convinced it adds substantial value if the obtained parameters too noisy to provide meaningful information (particularly meaningful null findings) in this small sample.

*A: Following the reviewer’s suggestions we now include a ‘model-agnostic’ approach to show a) trial-history effects of previous AV disparity on the current cross-modal bias which are captured by the effects on the causal prior in our model-based analysis and b) trial-history effects of the previous number of task-relevant signals on current numeric reports which are captured by the BCI model’s numeric prior mean (Supporting Fig. S6). We describe this finding in the Results (p. 13, l.23 ).*

*Trial-history effects, or in other terms serial dependencies, have not been investigated, to our knowledge, for the SIFI paradigm specifically and only in some studies for multisensory perception in general. We previously found that the recent history of visual reliability modulated the ventriloquist effect (Beierholm et al., 2020). Yet, previous AV disparity did not modulate the ventriloquist effect (Kayser et al., 2023). Further, multisensory perception adapts to more prolonged exposures to congruent or incongruent multisensory stimuli in ventriloquist and McGurk paradigms (Gau & Noppeney, 2016; Hong et al., 2022; Nahorna et al., 2012). However, trial-history effects in our SIFI paradigm are meaningful because they show that observers flexibly adapt to the statistical structure of recent multisensory stimuli on short time-scales, as predicted by theoretical accounts of serial dependencies in perception (Cicchini et al., 2024).*

*Additionally, we performed parameter recovery for the fitting approach and found that we could recover the BCI model’s causal prior and numeric prior mean as well as variance with high reliability and little bias (Supporting Fig. S14). We mention parameter recovery for this computational modelling analysis in the Methods (p. 31, l. 1).*

*We fully agree that one can conceive Bayesian models that incorporate recursive updating of priors, see e.g. our previous publication (Beierholm et al., 2020). However, such a modelling approach is computationally very costly to fit and therefore beyond the scope of the current study.*

*Because these trial-history effects in the SIFI are an important novel contribution in its own right, we prefer to keep these analyses. However, we would be able to remove this section if required by the reviewer or editor.*

Minor concerns

1. Most psychotic symptoms are not multisensory even though they can manifest in different modalities (e.g., even in patients who report visual and auditory hallucinations, integrated audiovisual hallucinations are rare). The focus on multisensory perception/integration should be more carefully justified. One clear strength is the ability to investigate different priors and hierarchical levels of inference. This is linked to point 1 above; cited studies (citations 30-36) focus on diagnosis comparisons rather than psychosis comparisons although the text does not make this distinction.

*A: The reviewer raises an interesting point. There are recent studies suggesting that multisensory hallucinations were far more prevalent than previously assumed (Dudley et al., 2023). Potentially, clinicians do not systematically elucidate the multisensory nature of hallucinations in routine psychiatric anamneses. We therefore think the jury is still out to decide whether hallucinations are of a multisensory nature. In fact, hallucinations may even arise because observers misbind several sensory signals that simply co-occur in time without being causally related. Such a mechanism may be particularly prominent when temporal binding windows are wider in psychosis. Following the reviewer’s suggestions, we have now discussed the possible multisensory nature and its relevance for understanding hallucinations in greater depth in an own paragraph in the Introduction (p. 3, l. 28). Further, we mention multisensory hallucinations as an important future research avenue in the Discussion (p. 18, l. 20).*

2. Average fit quality by group is shown. Is there substantial variability in the winning model by individual? If so, it would be important to show the proportions of winning models across groups and by psychosis severity.

*A: Overall, in the majority of individuals for the HC group (16/23) and the SCA group (11/17), the model averaging strategy with increasing sensory variances won the individual model comparison within participant (Supporting Tab. S2). To investigate the interesting question of modulation by psychosis severity, we plotted the individual model fit (i.e., relative BIC) of the five best models (i.e., with increasing sensory variances) as a function of psychosis severity (i.e. PANSS positive symptoms). However, Supporting Fig. S4 shows that the model-averaging model outperforms the competing models across the spectrum of psychosis severity. No pattern emerged that would suggest that any model type correlates with the severity of psychosis. We mention this observation in the Results (p. 11, l. 22).*

3. Comparisons of model fitted variances (visual, auditory, and prior) with specific symptoms of psychosis (hallucinations vs delusions) should be shown.

*A: In supplemental Tab. S3, we report correlations of self-reported hallucinatory experiences (LSHS) and paranoid thinking (PCL) with BCI model parameters. Yet, we have only found a borderline significant negative correlation of hallucinatory experiences and the auditory variances, indicating a stronger weighting of auditory information in case of stronger hallucinations. We now report this finding in the Results section (p. 12, l. 22). We further elaborate that this negative correlation of auditory variance together with the positive correlation of visual variance with positive symptoms suggests that individuals with higher psychosis severity put more weight on auditory than visual sensory information (p. 13, l. 10).*

**Reviewer #3:** Strengths of this paper are that it generally seems like a good next step in understanding whether and how integration of prior information and sensory inputs may be impacted in individuals with schizophrenia. The methods are sound, including useful details about the paradigm, analyses methods and statistics. It is also fairly clearly written and results interpreted reasonably. The study is novel in its use of psychophysical and EEG data as well as computational modeling to answer some research questions regarding causal inference. One major weakness is the small sample size. The findings supported some of the predictions, and these are reasonably well addressed and seem interesting. They stand to add somewhat to the body of knowledge advancing how we think about multisensory integration mechanisms from a Bayesian standpoint. However, not all predictions panned out, reducing impact of the paper. Further, while at a theoretical level, the explanation for the modeling is clear—how each parameter indexes decision processes needs to be clarified further. My comments/ queries are aimed at improving the clarity of the findings and placing within the broader context of clinical research as well.

*A: We thank the reviewer for her/his overall positive evaluation and took the reviewer’s suggestion to improve the rationale and explanations for our modelling approach and to further enhance the readability of the manuscript.*

1) It would be useful to briefly explain more about the modeling, especially the fused weighting, and setting up/ providing detailed rationale for the computational aspect in the introduction

*A: We agree that the modelling approach and its rationale needs more explication for less technically inclined readership. We have therefore now expanded this part in the introduction (p. 4, l.7; p. 5, l. 13) and specifically explain the different decision strategies including the stochastic fusion weighting in the Results (p. 10, l. 29ff) and Methods (p. 28, l. 11).*

2) ? It is mentioned that "the visual variance" correlated with positive symptoms--- This should be explained more in terms of which exact measure and what this correlation signifies in the context of overweighting priors. Were there any other symptom correlates in the SZ group. Any effect of MWT-B or VLMT scores?

*A: We agree that the correlation of visual variance and positive symptoms deserves more explanation. In terms of the weighting of priors versus sensory evidence, the correlation means that individuals with higher psychosis severity give less weight to visual sensory variances. Further, in new additional analyses (see comments to Reviewer 2 above), hallucination-severity (as measured by LSHS) correlated marginally but negatively with the auditory variance parameter (Supporting Tab. S3), which has implications for the relative weighting of prior, visual and auditory information as described in the Results (p. 13, l. 10): Individuals with higher psychosis severity put less weight on visual as compared to prior information, and also put more weight on auditory than visual sensory information.*

*Further, we correlated the BCI model parameters with neurocognitive tests (MWT-B, VLMT, TMT and Stroop) as suggested by the reviewers (Supporting Tab. S5). Interestingly, VLMT free recall performance was negatively correlated with the parameter that quantifies the linear increase in auditory and visual variances as well as the lapse rate. Lapse rate was also correlated with worse Stroop performance. TMT performance was positively correlated with the visual variance parameter. We elaborate these findings in the Discussion (p. 18, l. 1). In particular we discuss how SCZ’ lower numeric prior weight may also arise from differences in motivation and vigilance/cognitive performance.*

3) While the paradigm, analyses and results attempt to answer the question of sensory integration in terms of stimulus complexity , it would be useful to place these results in the context of other findings on overly strong priors e.g. Pavlovian conditioning and failures in perceptual updating and discussion on what implication the intricacies of multisensory integration have on specific symptoms—This would strengthen the discussion on specificity and clinical implications of this work

A: Thanks – we have now included additional discussions. In particular, we have discussed the relationship of our results showing largely intact weighting of prior versus multisensory information and intact dynamic updating of priors compared to previous reports of overweighting of priors versus unisensory information (e.g., Cassidy et al., 2018; Powers et al., 2017). As highlighted by reviewer 2, previous research into Pavlovian conditioning compared patients with and without auditory hallucinations, which contrasts with our sample that scored rather low on psychotic symptoms (discussed in p. 20, l. 26).

4) Limitations of the sample size and impact of the results/interpretability should be discussed

*A: Thanks – following the reviewer’s suggestions we now discuss the limits of our small sample size limits in greater depth (p. 20, l. 15; see responses to Rev. 1 and 2 above).*

**References**

Acerbi, L., Dokka, K., Angelaki, D. E., & Ma, W. J. (2018). Bayesian comparison of explicit and implicit causal inference strategies in multisensory heading perception. *PLoS computational biology*, *14*(7), e1006110.

Beierholm, U., Rohe, T., Ferrari, A., Stegle, O., & Noppeney, U. (2020). Using the past to estimate sensory uncertainty. *Elife*, *9*, e54172.

Cassidy, C. M., Balsam, P. D., Weinstein, J. J., Rosengard, R. J., Slifstein, M., Daw, N. D., Abi-Dargham, A., & Horga, G. (2018). A perceptual inference mechanism for hallucinations linked to striatal dopamine. *Current Biology*, *28*(4), 503-514. e4.

Cicchini, G. M., Mikellidou, K., & Burr, D. C. (2024). Serial Dependence in Perception. *Annual Review of Psychology*, *75*(1), 129–154. https://doi.org/10.1146/annurev-psych-021523-104939

de Gelder, B., Vroomen, J., Annen, L., Masthof, E., & Hodiamont, P. (2003). Audio-visual integration in schizophrenia. *Schizophr Res*, *59*(2–3), 211–218. https://doi.org/S0920996401003449 [pii]

Dudley, R., Watson, F., O’Grady, L., Aynsworth, C., Dodgson, G., Common, S., Day, B.-A., & Fernyhough, C. (2023). Prevalence and nature of multi-sensory and multi-modal hallucinations in people with first episode psychosis. *Psychiatry Research*, *319*, 114988. https://doi.org/10.1016/j.psychres.2022.114988

Gau, R., & Noppeney, U. (2016). How prior expectations shape multisensory perception. *Neuroimage*, *124*, 876–886.

Hong, F., Badde, S., & Landy, M. S. (2022). Repeated exposure to either consistently spatiotemporally congruent or consistently incongruent audiovisual stimuli modulates the audiovisual common-cause prior. *Scientific Reports*, *12*(1), 15532. https://doi.org/10.1038/s41598-022-19041-7

Kayser, C., Park, H., & Heuer, H. (2023). Cumulative multisensory discrepancies shape the ventriloquism aftereffect but not the ventriloquism bias. *PLOS ONE*, *18*(8), e0290461. https://doi.org/10.1371/journal.pone.0290461

Nahorna, O., Berthommier, F., & Schwartz, J.-L. (2012). Binding and unbinding the auditory and visual streams in the McGurk effect. *The Journal of the Acoustical Society of America*, *132*(2), 1061–1077. https://doi.org/10.1121/1.4728187

Noel, J.-P., Stevenson, R. A., & Wallace, M. T. (2018). Atypical audiovisual temporal function in autism and schizophrenia: Similar phenotype, different cause. *European Journal of Neuroscience*, *47*(10), 1230–1241.

Odegaard, B., & Shams, L. (2016). The brain’s tendency to bind audiovisual signals is stable but not general. *Psychological science*, *27*(4), 583–591.

Odegaard, B., & Shams, L. (2017). The relationship between audiovisual binding tendencies and prodromal features of schizophrenia in the general population. *Clinical Psychological Science*, *5*(4), 733–741.

Odegaard, B., Wozny, D. R., & Shams, L. (2016). The effects of selective and divided attention on sensory precision and integration. *Neuroscience letters*, *614*, 24–28.

Pearl, D., Yodashkin-Porat, D., Katz, N., Valevski, A., Aizenberg, D., Sigler, M., Weizman, A., & Kikinzon, L. (2009). Differences in audiovisual integration, as measured by McGurk phenomenon, among adult and adolescent patients with schizophrenia and age-matched healthy control groups. *Compr Psychiatry*, *50*(2), 186–192. https://doi.org/10.1016/j.comppsych.2008.06.004 S0010-440X(08)00089-8 [pii]

Powers, A. R., Mathys, C., & Corlett, P. R. (2017). Pavlovian conditioning–induced hallucinations result from overweighting of perceptual priors. *Science*, *357*(6351), 596–600.

Rohe, T., Ehlis, A.-C., & Noppeney, U. (2019). The neural dynamics of hierarchical Bayesian causal inference in multisensory perception. *Nature communications*, *10*(1), 1907. https://doi.org/10.1038/s41467-019-09664-2

Williams, L. E., Light, G. A., Braff, D. L., & Ramachandran, V. S. (2010). Reduced multisensory integration in patients with schizophrenia on a target detection task. *Neuropsychologia*, *48*(10), 3128–3136.

Wilson, R. C., & Collins, A. G. (2019). Ten simple rules for the computational modeling of behavioral data. *eLife*, *8*, e49547. https://doi.org/10.7554/eLife.49547

Zhou, H., Cai, X., Weigl, M., Bang, P., Cheung, E. F. C., & Chan, R. C. K. (2018). Multisensory temporal binding window in autism spectrum disorders and schizophrenia spectrum disorders: A systematic review and meta-analysis. *Neuroscience & Biobehavioral Reviews*, *86*, 66–76. https://doi.org/10.1016/j.neubiorev.2017.12.013
