# Supplementary material for: Multisensory perceptual and causal inference is largely preserved in medicated post-acute individuals with schizophrenia
Source: PLoS Biol. 2024 Sep 10;22(9):e3002790. doi: 10.1371/journal.pbio.3002790 (PMC11466413; doi:10.1371/journal.pbio.3002790)
Supplement: Attachment — Submitted filename: 2024_08_01_ResponseLetter_R2.docx [file pbio.3002790.s036.docx]

Thank you for your patience while we considered your revised manuscript "Computations and neural dynamics of audiovisual causal and perceptual inference in schizophrenia" for publication as a Update Article at PLOS Biology. This revised version of your manuscript has been evaluated by the PLOS Biology editors, the Academic Editor and the original reviewers.

Based on the reviews and on our Academic Editor's assessment of your revision, we are likely to accept this manuscript for publication, provided you satisfactorily address the remaining points raised by the reviewers. Please also make sure to address the following data and other policy-related requests.

*Response: We thank the editor and the reviewers for their efforts to review and evaluate our manuscript and their constructive comments. We have implemented all suggested changes and highlighted them in yellow in the revised manuscript. Further, we refer the editor and the reviewers to changes in the manuscript (page/line) in our responses.*

There is one point I forgot to mention in the previous decision letter: Can you please make sure to mention add to the competing interests statement that Uta Noppeney is an Editorial Board Member of PLOS Biology?

*Response: We have included this fact in the competing interest statement in the online submission system.*

* We would like to suggest a slightly different title to improve accessibility: "No evidence for impaired multisensory perceptual and causal inference in medicated post-acute individuals with schizophrenia"

*Response: We would like to follow the editor’s suggestion to make the title clearer, more accessible and more precise. Therefore, we suggest the following title “Multisensory perceptual and causal inference is largely preserved in medicated post-acute individuals with schizophrenia”. We feel that the formulation “No evidence for impaired…” is a kind of a double negation that is more difficult to read and understand. Further, Bayes factors provide evidence for largely equivalence of perceptual processes, given the small sample size. However, we are also fine the editor’s suggested title.*

* Please provide information in the methods section that the study has been conducted according to the principles expressed in the Declaration of Helsinki.

*Response: We have added this information of the Declaration of Helsinki in the Methods (p.24, l. 3)*

* Please also attend to the following points:

** Page 9 line 15, missing the word “bias”

** Page 15 line 34 “accuracyin”

** Fig 1C, the y-axis label “correlation” is not explained or described it seems.

*Response: Thanks for careful reading of our manuscript and spotting these errors, which we have now corrected.*

* Please include qualifiers where necessary to indicate that due to the small sample size, the evidence for a certain claim may be limited. Reviewer 1 mentions one of these statements in the abstract where this will be needed. Papers such as Gilles E. Gignac ⁎, Eva T. Szodorai. Effect size guidelines for individual differences researchers, Personality and individual differences (2016) and Button, K. S., Ioannidis, J. P., Mokrysz, C., Nosek, B. A., Flint, J., Robinson, E. S., & Munafo, M. R. (2013) might be useful for further discussing this issue, also considering that your power calculation is based on a large effect size (d=1) which may not be realistic given that effect sizes are usually rather small in this field (e.g., d=0.4).

*Response: We agree and thank you for pointing us to these important references to discuss our sample size limitations. We have included the small-sample-size limitation in the Abstract (p.2, l. 17) and qualify it throughout the manuscript in the Introduction (p. 6, l.1), Discussion (p. 21, l.5; p. 21, l. 16ff; p.22, l. 33) and Methods (p. 24, l. 9 ). Further, we discuss the potential overestimation of previous effects sizes in our power calculations using the suggested references in our limitations paragraph in the Discussion (p. 21, l. 24) and Methods (p. 24, l. 12).*

* DATA POLICY:

You may be aware of the PLOS Data Policy, which requires that all data be made available without restriction: <http://journals.plos.org/plosbiology/s/data-availability>. For more information, please also see this editorial: <http://dx.doi.org/10.1371/journal.pbio.1001797>

Note that we do not require all raw data. Rather, we ask that all individual quantitative observations that underlie the data summarized in the figures and results of your paper be made available in one of the following forms:

1) Supplementary files (e.g., excel). Please ensure that all data files are uploaded as 'Supporting Information' and are invariably referred to (in the manuscript, figure legends, and the Description field when uploading your files) using the following format verbatim: S1 Data, S2 Data, etc. Multiple panels of a single or even several figures can be included as multiple sheets in one excel file that is saved using exactly the following convention: S1_Data.xlsx (using an underscore).

2) Deposition in a publicly available repository. Please also provide the accession code or a reviewer link so that we may view your data before publication.

Regardless of the method selected, please ensure that you provide the individual numerical values that underlie the summary data displayed in the following figure panels as they are essential for readers to assess your analysis and to reproduce it: 1CD, 4, 5ABC and similar panels in the supplementary information.

NOTE: the numerical data provided should include all replicates AND the way in which the plotted mean and errors were derived (it should not present only the mean/average values).

Please also ensure that figure legends in your manuscript include information on where the underlying data can be found, and ensure your supplemental data file/s has a legend.

Please ensure that your Data Statement in the submission system accurately describes where your data can be found.

*Response: To adhere to the PLOS data policy (which we embrace), we have uploaded all raw behavioural and EEG data to a repository on Dryad (*[*https://datadryad.org/stash/share/brK4SQK2501hGCuxstOeeCGiJaWDMm50KO3RROjZd88*](https://datadryad.org/stash/share/brK4SQK2501hGCuxstOeeCGiJaWDMm50KO3RROjZd88) *; note this is a private sharing link for review and the repository will be made public upon acceptance) along with documentations and meta-data according to the FAIR principles. To avoid re-identifiability of individual participants, we only included limited demographic and clinical data (i.e., sex, age, diagnosis, PANSS positive, PANSS negative, PANSS general etc.). Further, we have uploaded source data underlying all manuscript figures as well as relevant supplemental figures (Figures S8 – S11) as supplemental information and reference the data in the figure legends.*

* CODE POLICY

Per journal policy, if you have generated any custom code during the course of this investigation, please make it available without restrictions. Please ensure that the code is sufficiently well documented and reusable, and that your Data Statement in the Editorial Manager submission system accurately describes where your code can be found.

Please note that we cannot accept sole deposition of code in GitHub, as this could be changed after publication. However, you can archive this version of your publicly available GitHub code to Zenodo. Once you do this, it will generate a DOI number, which you will need to provide in the Data Accessibility Statement (you are welcome to also provide the GitHub access information). See the process for doing this here: <https://docs.github.com/en/repositories/archiving-a-github-repository/referencing-and-citing-content>

*Response: We have included the code to present the audiovisual stimuli in the experimental paradigm, to fit the BCI model to the behavioural data and analyse the behavioural data in the repository on Dryad (*[*https://datadryad.org/stash/share/brK4SQK2501hGCuxstOeeCGiJaWDMm50KO3RROjZd88*](https://datadryad.org/stash/share/brK4SQK2501hGCuxstOeeCGiJaWDMm50KO3RROjZd88) *; note this is a private sharing link for review and the repository will be made public upon acceptance).*

As you address these items, please take this last chance to review your reference list to ensure that it is complete and correct. If you have cited papers that have been retracted, please include the rationale for doing so in the manuscript text, or remove these references and replace them with relevant current references. Any changes to the reference list should be mentioned in the cover letter that accompanies your revised manuscript.

*Response: Thanks - We have closely reviewed our reference list. We have added the references suggested by the editor (Button et al., 2013; Gignac et al., 2016) and another study investigating audiovisual binding in SCZ (Stevenson et al., 2017) in response to reviewer 1.*

------------------------------------------------------------------------

Reviewer remarks:

Reviewer #1: The authors have kindly answered our questions, clarified their methods and discussed the limitations of the study, and updated the manuscript accordingly.

Some aspects of the work are interesting and solid.

However, the limitations remain, particularly the fact that the authors mostly report negative findings with a very small sample (which is not well justified by a power analysis, and is poorly representative of a population of patients with schizophrenia as a whole, particularly in early or untreated states), using a design that had not been used before to show differences in SCZ (so not easily comparable to previous literature). The results concerning the numeric prior (the significance of which, unlike that of the p_common prior, was a bit unclear to me to start with) are now muddled/weaker (not significant) after inclusion of the 6 patients with schizo-affective disorder.

All in all, although I recognise how hard it is to get bigger samples of patients with schizohprenia and/or collecting data on different conditions (e.g. also varying SOAs to explore temporal windows), my opinion is that the contribution and significance of the presented results are limited (if the aim is to present them to a large/general audience). I don't think it is fair for example to express in such broad terms that the results " demonstrate that the neurocomputational mechanisms of multisensory perceptual and causal inference remain remarkably intact in medicated post-acute individuals with schizophrenia" (as written in the abstract) as the results may only hold for the chosen design (which doesn't explore differences in binding windows / replicate situations where differences have been observed) and may miss subtle differences that would be detected in a larger sample.

*Response: Thanks for these comments. To address the reviewer’s concerns, we have made the following changes:*

*1. We have emphasized the limitations of our small sample size throughout the entire manuscript, specifically in the Abstract (p.2, l. 17) and repeat it throughout the manuscript in the Introduction (p. 6, l.1), Discussion (p. 21, l.5; p. 21, l. 16ff; p.22, l. 33 ) and Methods (p. 24, l. 9 ). Further, we have down-tuned the conclusion of “remarkably intact” multisensory perception in SCZ to “largely preserved” or “largely similar” in the Title, the Abstract (p. 2, l. 16), Introduction (p. 5, l. 34), Results (p. 17, l. 1), and Discussion (p. 20, l. 28; p. 21, l. 15).*

*2. We have expanded the abstract to clarify that our study manipulated number of flashes and beeps rather than the temporal binding window (p. 2, l. 7).*

*3. We have now provided an in-depth discussion of a previous study that assessed Bayesian causal inference in SCZ in an audiovisual paradigm that manipulated audiovidual asynchrony (Noel et al., 2018) in the Results (p. 16, l. 3ff.) and Discussion (p. 22, l. 23). This study did not find any differences in causal prior between SCZ and controls either. Instead, the widening of the temporal integration window in SCZ was mainly attributed to changes in sensory uncertainty that can be measured more precisely when the AV asynchrony is manipulated. The reduction in sensory uncertainty may potentially explain the differences in EEG decoding of the visual estimates in our current study (p. 16; l. 12).*

Reviewer #2 (Guillermo Horga): The authors have been very responsive and addressed all my comments. This is a great paper and an important contribution to the literature.

*Response: We thank the reviewer for his positive evaluation and his previous insightful comments that significantly helped to improve the paper.*
